# Sox2 promotes tamoxifen resistance in breast cancer cells

**DOI:** 10.1002/emmm.201303411

**Published:** 2013-10-31

**Authors:** Marco Piva, Giacomo Domenici, Oihana Iriondo, Miriam Rábano, Bruno M Simões, Valentine Comaills, Inmaculada Barredo, Jose A López-Ruiz, Ignacio Zabalza, Robert Kypta, Maria d M Vivanco

**Affiliations:** 1 Cell Biology and Stem Cells Unit, CIC bioGUNEBilbao, Spain; 2 Department of Pathology, Galdakao-Usansolo HospitalGaldakao, Spain; 3 Servicio de Radiodiagno´stico PreteimagenBilbao, Spain; 4 Department of Surgery and Cancer, Imperial College LondonLondon, UK

**Keywords:** breast cancer, Sox2, stem cells, tamoxifen resistance, wnt signalling

## Abstract

Development of resistance to therapy continues to be a serious clinical problem in breast cancer management. Cancer stem/progenitor cells have been shown to play roles in resistance to chemo- and radiotherapy. Here, we examined their role in the development of resistance to the oestrogen receptor antagonist tamoxifen. Tamoxifen-resistant cells were enriched for stem/progenitors and expressed high levels of the stem cell marker Sox2. Silencing of the *SOX2* gene reduced the size of the stem/progenitor cell population and restored sensitivity to tamoxifen. Conversely, ectopic expression of Sox2 reduced tamoxifen sensitivity *in vitro* and *in vivo*. Gene expression profiling revealed activation of the Wnt signalling pathway in Sox2-expressing cells, and inhibition of Wnt signalling sensitized resistant cells to tamoxifen. Examination of patient tumours indicated that Sox2 levels are higher in patients after endocrine therapy failure, and also in the primary tumours of these patients, compared to those of responders. Together, these results suggest that development of tamoxifen resistance is driven by Sox2-dependent activation of Wnt signalling in cancer stem/progenitor cells.

## Introduction

Breast cancer is the most common female cancer and approximately 70–75% of cases express oestrogen receptor alpha (ERα). Tamoxifen, an oestrogen antagonist in the breast, has been the standard endocrine therapy for women with ERα-positive breast cancer for many years and remains so for premenopausal and a substantial number of postmenopausal patients (Jordan & O'Malley, 2007). In many cases, however, resistance to endocrine therapy develops, although ERα expression is maintained in most tumours that acquire resistance (Ali & Coombes, 2002).

The potential mechanisms underlying this resistance to endocrine therapy involve ER-coregulatory proteins and cross-talk between the ER pathway and other growth-factor signalling networks (Osborne *et al,* 2005). A growing body of evidence is accumulating supporting the hypothesis that cancer stem cells, or tumour-initiating cells, drive and maintain many types of human malignancies (Diehn *et al,* 2009). The cancer stem cell hypothesis has shed new light on the development of resistance to therapy, proposing that there exists a pool of malignant cells with stem/progenitor cell properties and increased capacity to resist common chemotherapeutic treatments, compared to their more differentiated non-tumourigenic counterparts, and therefore responsible for tumour recurrence after treatment (Reya *et al,* 2001). Breast cells with the phenotype CD44^+^CD24^−/low^lineage^−^ isolated from metastatic pleural effusions by fluorescence activated cell sorting (FACS) are highly enriched for tumour-initiating cells (Al-Hajj *et al,* 2003). Importantly, the CD44^+^CD24^−/low^ cell population increases in size after chemotherapy and is associated with enhanced ability to form mammospheres, suggesting that these cells are more resistant to treatment (Li *et al,* 2008). In addition, normal and cancer breast epithelial cells with increased aldehyde dehydrogenase activity (ALDH) show stem/progenitor cell properties*in vitro* and *in vivo* and are associated with poor clinical outcome (Ginestier *et al,* 2007). Finally, poorly differentiated breast tumours contain a higher proportion of cancer stem cells than well-differentiated cancers (Pece *et al,* 2010).

Previously, we observed that oestrogen reduces the pool of breast stem cells while tamoxifen has the opposite effect (Simoes *et al,* 2011). The relevance of the increase in the proportion of cancer stem cells upon tamoxifen treatment is intriguing in the context of the development of tamoxifen resistance in breast cancer patients. Furthermore, normal and cancer stem cells share phenotypes that may reflect the activity of common signalling pathways, such as high expression of *NANOG*, *OCT4* and *SOX2*, which is reduced by oestrogen (Simoes *et al,* 2011). In breast tumours, an embryonic stem cell (ES)-like signature characterized by activation of targets of Nanog, Oct4 and Sox2 is associated with high-grade ER-negative tumours and with aggressive tumour behaviour (Ben-Porath *et al,* 2008), supporting the possibility that ES genes contribute to the stem cell-like phenotype found in many tumours.

Here, we present evidence that Sox2, a transcription factor that is key in maintaining pluripotent properties of stem cells, is a crucial player in the development of resistance to tamoxifen in ER-positive breast cancer cells. Sox2 overexpression increases the proportion of breast cancer stem/progenitor cells by activating the Wnt signalling pathway, thereby rendering the cells insensitive to the growth inhibitory effects of tamoxifen. These findings, together with the observation that Sox2 levels are elevated in the primary tumours of patients that do not respond to endocrine therapy, suggest that Sox2 could represent a prognostic factor for development of resistance to tamoxifen and that Wnt signalling may be an attractive therapeutic target in these patients.

## Results

### Increased tumourigenicity during the development of tamoxifen resistance compromises ER transcriptional activity

**Figure 1:**
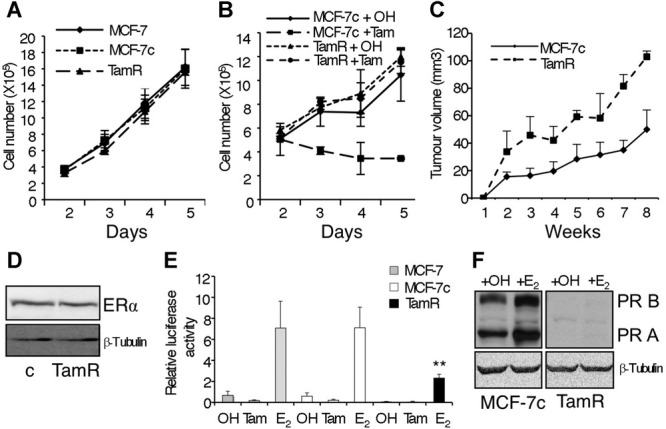
Characterization of MCF-7TamR cells. A  Proliferation assay of MCF-7 (wt), MCF-7c (control) and MCF-7TamR (tamoxifen resistant) cells (*n* = 3). B  Proliferation assay of MCF-7c and MCF-7TamR cells treated with ethanol (OH) or 5 × 10^−7^ M tamoxifen (Tam) (*n* = 3). C  Tumour growth curve of MCF-7c and MCF-7TamR cells implanted s.c. in athymic mice in the presence of an exogenous slow release, oestrogen implant (*n* = 5 mice/group). D  Western blot analysis of ERα expression in MCF-7c (c) and MCF-7TamR cells. E  MCF-7 (grey bars), MCF-7c (white bars) and MCF-7TamR (black bars) cells were transfected with a reporter plasmid containing three copies of a consensus ERE driving a luciferase reporter in the presence of the carrier ethanol (OH) or 5 × 10^−7^ M tamoxifen (Tam) or 10^−8^ M oestrogen (E_2_). In all transfections, β-galactosidase activity was used to control for transfection efficiency (*n* = 5) **p = 0.007 by *t*-test. F  Progesterone receptor expression in control (MCF-7c) and resistant (TamR) cells by Western blot analysis.

The development of tamoxifen resistance in breast cancer cells was used as a model for the acquisition of resistance to oestrogen antagonists that occurs in breast cancer patients. The oestrogen sensitive MCF-7 breast cancer cell line was cultured in the presence of tamoxifen or, in parallel, with the carrier ethanol. Initially, cell growth rates were very much reduced in the presence of tamoxifen, but eventually cells adapted to the new environment leading to two new sub-lines: MCF-7TamR (resistant to tamoxifen treatment) and control MCF-7c cells. The control MCF-7c cells are indistinguishable from the parental MCF-7 cells with respect to their proliferation capacity in normal growth medium, which is also similar to the MCF-7TamR cells (Fig 1A). As expected, control cell proliferation was reduced by tamoxifen, while MCF-7TamR cells grew at a similar rate, independently of the presence of tamoxifen (Fig 1B). Subcutaneous transplantation of MCF-7TamR cells to athymic mice led to larger and faster growing tumours compared to the parental MCF-7 cells, indicating their increased tumourigenic potential (Fig 1C). Immunoblot analysis revealed that MCF-7TamR cells remain ERα positive (ERα will be referred to as ER), like parental MCF-7 cells (Fig 1D). Despite constant levels of ER expression, ER transcriptional activity was lower in MCF-7TamR cells than in control cells in the context of a consensus oestrogen response element (Fig 1E). In addition, expression of the progesterone receptor (PR), a well-known ER target gene, was strongly reduced in MCF-7TamR cells (Fig 1F). These results indicate that MCF-7TamR cells are more tumourigenic than parental MCF-7 cells and, although they express comparable levels of ER, its transcriptional activity is reduced in MCF-7TamR cells.

### Tamoxifen resistant cells express high levels of SOX2

**Figure 2:**
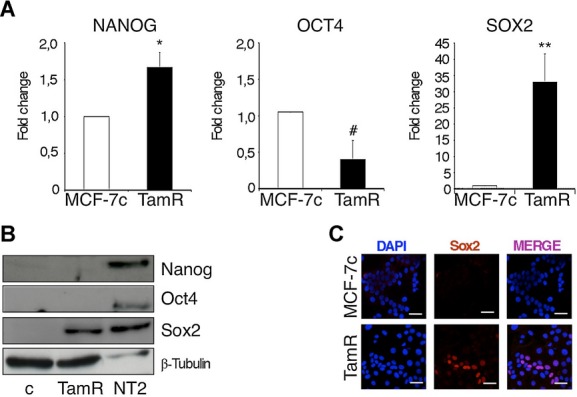
MCF-7TamR cells express high levels of SOX2. A  Transcript levels of NANOG, OCT4 and SOX2 in adherent MCF-7c and MCF-7TamR (TamR) cells were quantified by real-time PCR and presented as fold induction with MCF-7c value set as 1 (*n* = 5) *p = 0.03, ^#^*p* = 0.023, **p = 0.004 by *t*-test. B  Immunoblots of Nanog, Oct4, Sox2 and β-tubulin (loading control) in MCF-7c and MCF-7TamR cells. NTera2/D1 (NT2) cells were used as positive control for the expression of the stem cell markers. C  Immunofluorescence analysis of Sox2 expression in MCF7c and MCF-7TamR cells. Scale bar = 40 μm.

We have recently shown that the embryonic stem cell markers *NANOG*, *OCT4* and *SOX2* are expressed in normal breast stem cells and at higher levels in breast tumour cells and that their expression is reduced during cell differentiation (Simoes *et al,* 2011). Therefore, we wished to determine whether the expression of *NANOG*, *OCT4* and *SOX2* is differentially modulated during development of tamoxifen resistance. Real-time PCR analysis showed that the level of expression of *SOX2* was 30-fold higher in MCF-7TamR cells than in control cells. In comparison, the expression levels of *NANOG* and *OCT4* were not strongly affected (Fig 2A). In agreement with the PCR data, the levels of Sox2 protein were also clearly elevated in MCF-7TamR cells, although they were lower than Sox2 levels in undifferentiated human embryonal carcinoma stem cells (NTera2/D1 cell line) (Fig 2B). Finally, immunofluorescence analysis showed that Sox2 was strongly expressed in 20–30% of the MCF-7TamR cells (Fig 2C). These results indicate that Sox2 expression levels are higher in a subpopulation of tamoxifen resistant cells than in parental breast cancer cells.

### Development of tamoxifen resistance in breast cancer cells increases the proportion of stem/progenitor cells

**Figure 3:**
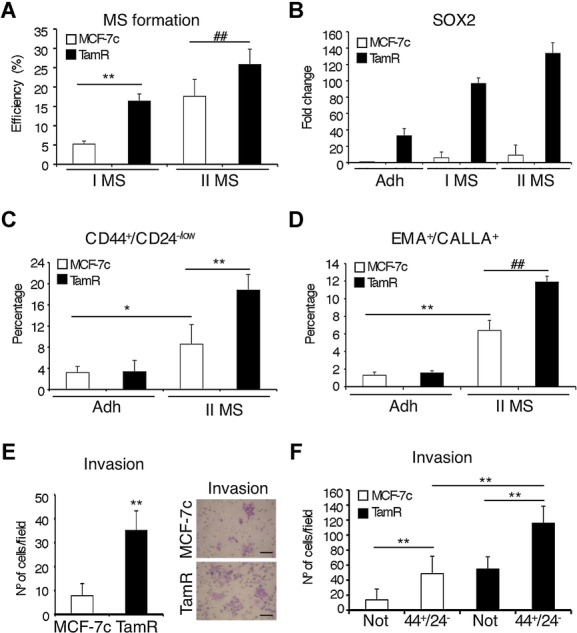
MCF-7TamR cells contain a higher proportion of stem cells than parental cells. A  Primary (I MS) and secondary (II MS) mammosphere efficiency formation from MCF-7c and MCF-7TamR cells represented as the percentage of mammospheres formed (*n* = 5) **p = 0.003, ^##^*p* = 0.008 by *t*-test. B  SOX2 mRNA expression levels in MCF-7c and MCF-7TamR cells grown in adherent (Adh) or mammosphere (I MS and II MS) cultures were quantified by real-time PCR (*n* = 3). C, D  Flow cytometry analysis of (C) CD44^+^/CD24^−/low^ (*n* = 5, *p = 0.028 **p = 0.0045 by *t*-test) and (D) EMA^+^/CALLA^+^ (*n* = 3, **p = 0.0021, ##*p* = 0.0028 by *t*-test) stem cell populations in MCF-7c and MCF-7TamR cells cultured as adherent cells (Adh) or as secondary mammospheres (II MS), represented as the percentage of cells with the indicated phenotype within the total population. E  Matrigel invasion assay was performed using adherent MCF-7c and MCF-7TamR cells. The photographs on the right show a representative field (*n* = 3) **p = 0.001 by *t*-test. Scale bar = 100 μm. F  Matrigel invasion assay was performed using secondary mammospheres from MCF-7c and MCF-7TamR cells FACS-sorted to isolate CD44^+^CD24^−/low^ (44^+^24^−^) stem cells and the remaining cell population lacking CD44^+^z24^−/low^ cells (Not) (*n* = 3) **p < 0.001 by *t*-test.

To monitor whether the self-renewal capacity of the breast stem/progenitor cell population was affected by the development of tamoxifen resistance, the efficiency of mammosphere formation was examined. We observed that MCF-7TamR cells formed a significantly higher number of primary and secondary mammospheres than the control cells, indicating increased self-renewal capacity (Fig 3A). Quantitative PCR analysis showed that *SOX2* expression is higher in primary and secondary mammospheres than in cells grown in adherent differentiating cultures (Fig 3B), mirroring the numbers of spheres formed. Furthermore, MCF-7TamR cells expressed higher levels of *SOX2* than control cells, both in adherent and suspension conditions (Fig 3B). In contrast, although the expression of *NANOG* and *OCT4* was higher in mammospheres than in adherent cultures, as previously shown (Simoes *et al,* 2011), there were no significant differences between their levels of expression in MCF-7TamR and control cells (supplementary Fig 1A). These findings suggest that Sox2 is relevant to the development of resistance to tamoxifen.

In breast carcinomas, a cell population with the phenotype CD44^+^CD24^−/low^ has been shown to be enriched for tumourigenic stem/progenitor cells (Al-Hajj *et al,* 2003). FACS analysis showed a significant increase in the proportion of CD44^+^CD24^−/low^ cells in secondary mammospheres when compared to cells grown in adherent cultures, both in control and MCF-7TamR cells, although this increase was significantly stronger in tamoxifen resistant cells than in MCF-7c cells (Fig 3C and supplementary Fig 1B and D). In addition, we determined the percentage of EMA^+^CALLA^+^ cells, since sorting for this cell population has been shown to enrich for normal (Clayton *et al*, 2004) and cancer (Simoes *et al,* 2011) breast stem/progenitor cells. Indeed, the proportion of EMA^+^CALLA^+^ cells was higher in mammospheres than in adherent cultures and was highest in MCF-7TamR cells (Fig 3D and supplementary Fig 1C). These results suggest that MCF-7TamR cells contain a higher percentage of stem/progenitor cells than parental breast cancer cells.

Previous studies have shown an association between the CD44^+^CD24^−/low^ phenotype and invasion (Sheridan *et al,* 2006). Consistent with this, MCF-7TamR cells exhibited an increased invasion capacity through Matrigel, compared with control cells (Fig 3E). Isolated stem cells with the phenotype CD44^+^CD24^−/low^ displayed a significant increase in their capacity to migrate in Transwell assays (supplementary Fig 1E), and particularly to invade through Matrigel (Fig 3F), compared to cells of the reverse phenotype. Furthermore, this increase was more evident in stem cells isolated from MCF-7TamR than more differentiated cells (Fig 3F). These results show that tamoxifen resistant cells possess higher invasion capacity than parental breast cancer cells and that this phenotype correlates with the proportion of CD44^+^CD24^−/low^ cells.

### Inverse correlation of ER and Sox2 protein levels in tamoxifen resistant breast cancer cells

**Figure 4:**
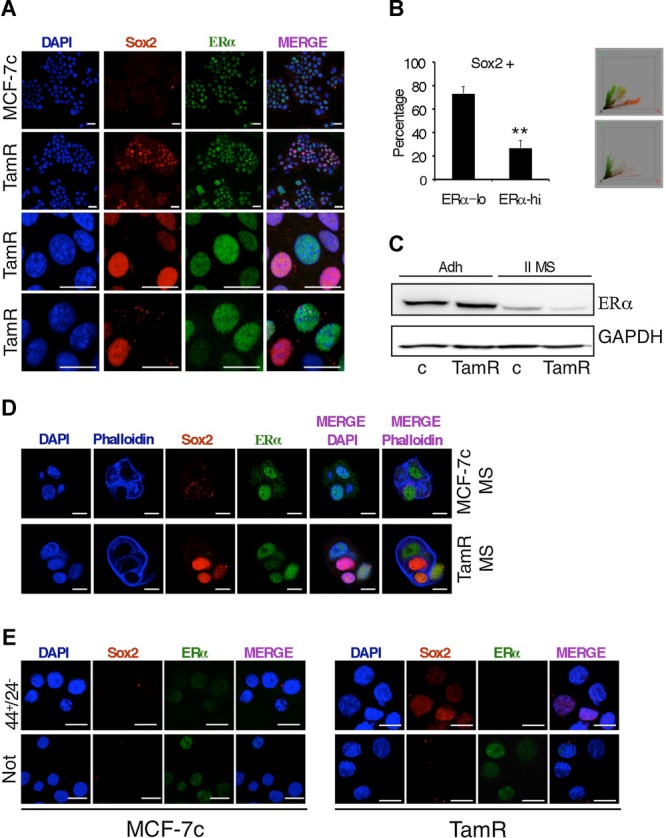
Inverse association between ER and Sox2 expression in MCF-7TamR cells. A  Coexpression of SOX2 and ER was visualized by immunofluorescence in MCF-7c and MCF-7TamR cells grown as adherent cells (scale bar = 40 μm) and, B  as secondary mammospheres at day 4 (Scale bar = 20 μm). C  Left, percentage of adherent MCF-7TamR cells that were Sox2-positive and that expressed low (lo) or high (hi) ER levels (*n* = 3) **p = 0.0011 by *t*-test. Right, 2 representative plots obtained with ImageJ 3d colour inspector analysis, in green and in red, ER and Sox2 positivity, respectively. D  Western blot analysis of ER expression levels in MCF-7c and MCF-7TamR cells grown as adherent cells (Adh) or as secondary mammospheres (II MS). GAPDH was used as a loading control. E  Immunofluorescence analysis of Sox2 and ER expression in FACS-sorted CD44^+^CD24^−/low^ (44^+^24^−^) stem cells and the rest of the cell population lacking CD44^+^CD24^−/low^ cells (Not). Scale bar = 30 μm.

Breast stem/progenitor cells lack or express low levels of ER (Clayton *et al,* 2004; Liu *et al,* 2008). To examine the relationship between ER and Sox2 expression, immunofluorescence analysis was performed. As shown previously, Sox2 expression was difficult to detect in control MCF-7c cells, whereas it was expressed in around 20–30% of MCF-7TamR cells (Fig 2C and 4A). Although MCF-7 cells express ER, they do so at quite variable levels and cells with the highest levels of ER expression did not express Sox2, while cells with the highest levels of Sox2 displayed the lowest levels of ER (Fig 4A). When 300 cells from random fields from three independent experiments were counted, approximately 30% of MCF-7TamR cells were found to express Sox2, and of those Sox2-positive cells, 70% expressed low levels of ER and 30% expressed a high level of ER (Fig 4B). Representative plots obtained using ImageJ 3D colour inspector analysis further demonstrate the segregation between ER and Sox2 expressing cells (Fig 4B). Similar results were obtained using the ER-positive breast cancer cell lines T-47D and ZR-75-1 (supplementary Fig 2A and B).

To enrich for stem/progenitor cells, we cultured MCF-7TamR cells as mammospheres. Western blot analysis showed that ER expression was reduced in mammosphere cultures (Fig 4C). Moreover, Sox2-positive cells generally did not express ER in mammospheres, as shown by immunofluorescence (Fig 4D and supplementary Fig 2C). Furthermore, FACS sorted CD44^+^CD24^−/low^ cells expressed barely detectable levels of ER, both when isolated from control and MCF-7TamR cultures (Fig 4E). As expected, Sox2 was detected at the highest levels in the CD44^+^CD24^−/low^ cells isolated from MCF-7TamR mammospheres and was consistently absent from the population lacking this phenotype (Fig 4E). These results indicate that the most undifferentiated stem cell-like Sox2-positive cells do not express ER. These findings support the association between the CD44^+^CD24^−/low^ phenotype of breast cancer tamoxifen-resistant cells and Sox2 expression and suggest that cells expressing high levels of Sox2 will be more resistant to tamoxifen.

### Alteration of SOX2 expression levels affects stem cell content and tamoxifen sensitivity

**Figure 5:**
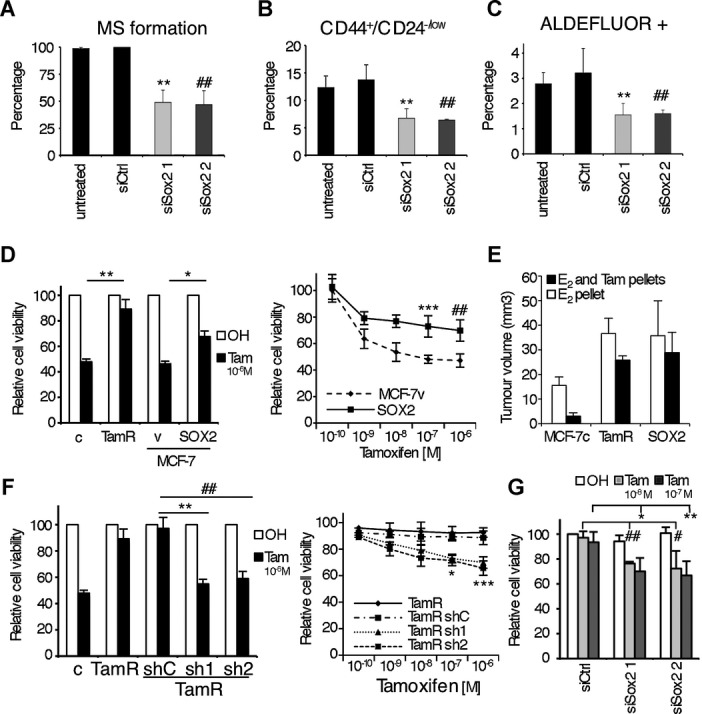
Alteration of Sox2 expression levels affects stem cell properties. A  Mammosphere formation assay of MCF-7TamR cells transfected with siRNA. Values obtained with scramble siRNA are set as 100% (*n* = 3) **p = 0.01, ^##^*p* = 0.009 by *t*-test. B  CD44^+^CD24^−/low^ stem cell population analysis of primary mammospheres (*n* = 3) **p = 0.011, ^##^*p* = 0.01 by *t*-test and C  ALDEFLUOR assays in adherent cells, were all performed using MCF-7TamR cells transfected with 2 different Sox2 siRNA sequences (siSox2 1 and 2) as well as a control siRNA sequence (siCtrl) (*n* = 3) **p = 0.002, ^##^*p* = 0.009 by *t*-test. D  Viability analysis by crystal violet (left) of MCF-7c (c), MCF-7TamR (TamR) cells and MCF-7 cells stably overexpressing Sox2 (Sox2) and control MCF-7v (v) cells (*n* = 5) **p = 0.008, *p = 0.02 by *t*-test and (right) MTT assays of MCF-7SOX2 (SOX2) and control MCF-7v (v) cells growing in presence of increasing concentrations of tamoxifen (from 10^−10^ M to 10^−6^ M) (*n* = 5) ***p = 0.008, ^##^*p* = 0.007 by *t*-test. E  Tumour size 3 weeks after s.c. implantation of MCF-7c, MCF-7TamR and MCF-7SOX2 cells in athymic female mice in the presence of an exogenous slow oestrogen supplement and with or without a tamoxifen pellet (*n* = 5 mice/group). F  Viability analysis by (left) crystal violet (*n* = 5, **p = 0.003, ^##^*p* = 0.02 by *t*-test) and (right) MTT assays (*n* = 5, *p = 0.004, ***p = 0.0008 by *t*-test) of MCF-7c (c), MCF-7TamR (TamR) and MCF-7TamR cells stably transfected with shRNA against Sox2 (sh1 and sh2) and control (shC), growing in the presence of vehicle (ethanol, OH) or tamoxifen. G  Viability analysis by crystal violet and of BT474 cells transfected with a control siRNA sequence (siCtrl) and two different Sox2 siRNA sequences (siSox2 1 and 2) growing in the presence of vehicle (ethanol, OH) or tamoxifen at different concentrations (10^−8^ M, ##*p* = 0.009, #*p* = 0.017, 10^−7^ M, *p = 0.044, **p = 0.002 by *t*-test, as indicated, *n* = 3).

To determine the relevance of Sox2 expression to the stem cell phenotype, we reduced endogenous *SOX2* levels in MCF-7TamR cells using siRNA. Transfection of two different siRNA sequences directed against Sox2 resulted in a strong reduction of Sox2 expression, as detected by immunofluorescence, while a control sequence did not have any effect (supplementary Fig 3A). Downregulation of Sox2 expression led to a significant inhibition of mammosphere formation by MCF-7TamR cells (Fig 5A), and a significant reduction in the percentage of cells with the phenotype CD44^+^CD24^−/low^ (Fig 5B and supplementary Fig 3C). Furthermore, MCF-7TamR cells contained a significantly higher population of ALDEFLUOR-positive cells, this is with high ALDH activity (Ginestier *et al,* 2007), than control cells (supplementary Fig 3B) and these cells expressed higher ALDH1A3 and Sox2 levels than the ALDEFLUOR-negative subpopulation (supplementary Fig 3E). Therefore, the ALDEFLUOR-positive subpopulation was also specifically reduced by Sox2 siRNA, while it was not affected by a control siRNA (Fig 5C and supplementary Fig 3D).

We have previously shown that stable overexpression of Sox2 in MCF-7 cells increases the frequency of stem cells and their capacity for invasion, properties associated with tumourigenesis and poor prognosis (Simoes *et al,* 2011). Importantly, breast cancer cells overexpressing Sox2 showed an enhanced resistance to the antiproliferative effects of tamoxifen treatment *in vitro* (Fig 5D) and *in vivo* (Fig 5E). In contrast, overexpression of Nanog or Oct4 did not affect sensitivity to tamoxifen (supplementary Fig 3F). On the other hand, stable downregulation of Sox2 expression (supplementary Fig 3G) in MCF-7TamR cells rendered them more sensitive to tamoxifen (Fig 5F). This reduction in cell viability under tamoxifen treatment was due to increased apoptosis rather than cell cycle arrest (supplementary Fig 4A and B, respectively). Finally, we wished to evaluate whether Sox2 levels are relevant to tamoxifen sensitivity in other tamoxifen resistance models. Reduction of endogenous Sox2 expression levels in tamoxifen resistant BT-474 cells (supplementary Fig 3H) was sufficient to increase their sensitivity to tamoxifen (Fig 5G). In addition, we developed breast cancer T47D cells resistant to tamoxifen (T47DTamR) by exposing them to tamoxifen for a period of over 8 months. Development of resistance to tamoxifen led to elevated endogenous Sox2 levels and decreased PR expression (supplementary Fig 4C), as observed in MCF-7TamR cells. Furthermore, Sox2 downregulation by specific siSox2 sequence in T47DTamR cells significantly reduced their resistance to tamoxifen (supplementary Fig 4D). Collectively, these results demonstrate that Sox2 plays a key role in the maintenance of the increased stem cell population associated with the development of tamoxifen resistance.

### Elevated SOX2 expression levels correlate with poor prognosis and development of recurrence in breast cancer patients

**Figure 6:**
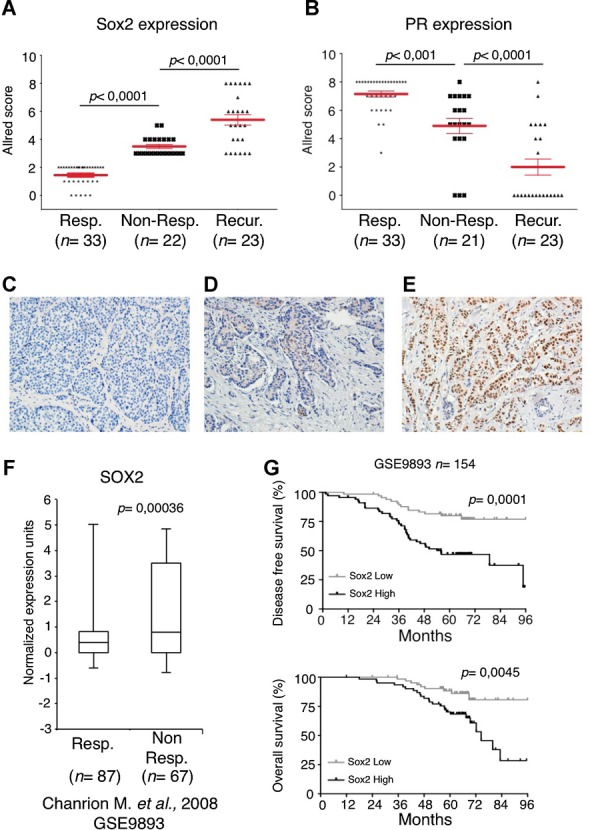
Sox2 expression increases during the development of tamoxifen resistance in breast cancer patients. A  Allred score for Sox2 (A) and PR staining (B). Patients with responder primary tumours (*n* = 33), namely those that responded to tamoxifen treatment (Resp.); patients with non-responder primary tumours (*n* = 22) (Non-Resp.) and their recurrent tumours after therapy failure (Recur.). *p*-values were calculated by Bonferroni multiple comparable test. C–E  Examples of Sox2 staining in (C) a tumour responsive to treatment, (D) a primary tumour not responsive to therapy and (E) a recurrent lesion from the matched primary tumour in (D). F  Correlation between Sox2 expression and recurrence in ER-positive tamoxifen treated breast cancer patients. Box plot from the study indicated (Chanrion et al, 2008) is shown. The *y*-axis shows normalized expression units. Data are median centered and the 25th–75th percentiles are indicated by the closed box. The numbers of breast carcinoma samples present are shown in parentheses and GEO accession numbers are indicated. Resp and Non Resp. tumours as above. G  The association between Sox2 expression levels and disease free survival (top) and overall survival (bottom) was evaluated by Kaplan–Meier analysis. *p*-values were calculated by Cox proportional hazards regression analyses. The Sox2 low group was defined by expressing lower Sox2 levels than the median of all patients in the study, and the rest of the patients belonged to the Sox2 high group.

The above findings raised the possibility that Sox2 expression may be altered during development of tamoxifen resistance in patients. To test this hypothesis, we examined a series of ER-positive breast tumour samples from 55 patients that had received tamoxifen therapy and a minimum of 6-year follow-up was available (supplementary Table S1). The cohort included patients for whom the endocrine therapy was successful (responders) and the tumour had not returned over a period of 8 years (*n* = 33 patients) and in which Sox2 was weakly expressed in a low percentage of cells (Allred score ≤2) (Fig 6A). In contrast, in non-responder patients all primary tumours stained positive for Sox2 (22 patients); furthermore, there was a significant increase in Sox2 expression in the recurrent lesions (26 samples, since four of them recurred twice), compared with the matched primary tumours. Representative photographs of tumour samples with negative (Fig 6C), moderate (Fig 6D) and strong (Fig 6E) Sox2 staining are shown. Interestingly, elevated Sox2 levels significantly correlated with decreased PR expression and increased histological grade during development of tamoxifen resistance (Fig 6B and supplementary Table S1).

In addition, in order to determine whether the expression of Sox2 has prognostic potential in breast cancer patients treated with tamoxifen, we analysed publicly available patient data sets (GSE9893, GSE12093 and GSE1379) where ER-positive patients (*n* = 154, 132 and 54, respectively) had been treated with tamoxifen therapy and a minimum of 5-year follow-up data are available. High Sox2 levels significantly correlated with poor overall survival and disease free survival (Fig 6F and G, and supplementary Fig 5). Taken together, these findings suggest that Sox2 expression in primary ER-positive tumours may be a clinical prognostic biomarker for tamoxifen resistance.

### Increased expression of Sox2 leads to activation of Wnt signalling

**Figure 7:**
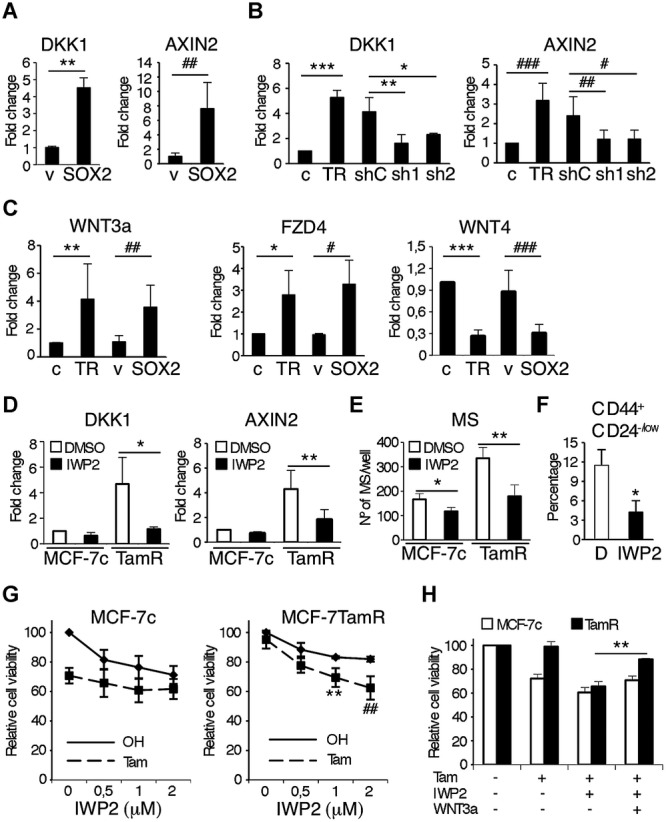
Sox2 overexpression leads to the activation of Wnt signalling. A  DKK1 and AXIN2 mRNA expression levels in MCF-7v (v) and MCF-7SOX2 cells grown in adherent conditions (*n* = 5) **p = 0.002, ^##^*p* = 0.002 by *t*-test. B  DKK1 (***p = 0.0001, **p = 0.009, *p = 0.042 by *t*-test) and AXIN2 (^###^*p* = 0.009,^##^*p* = 0.043, ^#^*p* = 0.029 by *t*-test) mRNA expression levels in MCF-7c (c) and MCF7TamR (TR) cells and MCF-7TamR cells stably transfected with 2 different Sox2 shRNA sequences (sh1 and sh2) as well as a control shRNA sequence (shC) grown in adherent conditions (*n* = 5). C  Wnt-3a (**p = 0.008, ^##^*p* = 0.0056 by *t*-test), FZD4 (*p = 0.021, ^#^*p* = 0.016 by *t*-test) and WNT4 (***p = 0.0001, ^###^*p* = 0.0015 by *t*-test) mRNA expression levels in MCF-7c (c), MCF7TamR (TR), MCF-7v (v) and MCF-7SOX2 cells grown in adherent conditions (*n* = 5). D  DKK1 and AXIN2 mRNA expression levels in MCF-7c (c) and MCF7TamR (TR) cells treated for 48 h with 1 μM of IWP-2 or the vehicle (DMSO) (*n* = 3) *p = 0.012, **p = 0.017 by *t*-test. E  Mammosphere formation assay from MCF-7c and MCF-7TamR (TamR) cells growing in presence of 1 μM of IWP-2 or the vehicle (DMSO) (*n* = 3) *p = 0.036, **p = 0.002 by *t*-test. F  Flow cytometry analysis of the CD44^+^/CD24^−/low^ stem cell population in MCF-7TamR cells grown as mammospheres in presence of 1 μM of IWP-2 or the vehicle (D) (*n* = 3) *p = 0.045 by *t*-test. G  Cell viability analysis by MTT assay of MCF-7c (left) and MCF-7TamR (right) cells growing in the presence of increasing concentrations of IWP-2 (from 0.5 μM to 2 μM) and in presence or absence of 10^−7^ M tamoxifen (*n* = 5) **p = 0.002, ^##^*p* = 0.0011 by *t*-test. H  Cell viability analysis by MTT assay of MCF-7c and MCF-7TamR cells growing in the presence or absence of 10^−7^ M tamoxifen, 1 μM IWP-2 and 100 ng/ml recombinant Wnt-3a as indicated (*n* = 3) **p = 0.001.

To unravel the mechanism of action of Sox2 in adherent and mammosphere cultures of breast cancer cells, we performed global gene expression analysis. Comparison of the expression profiles of Sox2-overexpressing cells with parental MCF-7 cells in adherent and suspension culture conditions highlighted the relevance of the Wnt signalling pathway in Sox2-overexpressing cells (supplementary Fig 6A and B). DKK1 and AXIN2, two known Wnt target genes, were among the most significant differentially expressed genes in Sox2-overexpressing cells. Their increased levels of expression due to Sox2 overexpression were confirmed by qPCR (Fig 7A). Inhibition of Sox2 expression in tamoxifen resistant cells using two different Sox2 shRNAs was sufficient to reduce DKK1 and AXIN2 expression (Fig 7B). Furthermore, Wnt-3a and its receptor FZD4, also identified by microarray analysis, were induced both in tamoxifen resistant cells and in Sox2-overexpressing cells, while expression of WNT4, which inhibits Wnt/β-catenin signalling (Elizalde *et al,* 2011), was reduced (Fig 7C). To determine if activation of the Wnt signalling pathway in these cells was mediated by an autocrine pathway, we used the small-molecule porcupine inhibitor IWP-2, which blocks Wnt secretion (Chen *et al,* 2009). Addition of IWP-2 reduced DKK1 and AXIN2 expression in tamoxifen resistant breast cancer cells (Fig 7D). Furthermore, IWP-2 significantly reduced the cancer stem cell population in tamoxifen resistant cells, as confirmed by reduced capacity for mammosphere formation (Fig 7E) and a reduction in the percentage of CD44^+^CD24^−/low^ cells (Fig 7F). Crucially, IWP-2 restored tamoxifen sensitivity to MCF-7TamR cells (Fig 7G) and this effect of IWP-2 was prevented upon addition of exogenous purified Wnt-3a protein (Fig 7H). These findings indicate that autocrine Wnt signalling protects breast cancer cells from the anti-proliferative effects of tamoxifen.

## Discussion

Development of resistance to tamoxifen remains an important clinical problem. Here we demonstrate that tamoxifen resistant MCF-7 cells express higher levels of Sox2 than parental breast cancer cells. In addition, tamoxifen resistant cells contain a higher proportion of cancer stem/progenitor cells and are more invasive than parental cells. There is an inverse correlation between ER and Sox2 expression in breast cancer cells and an association between the CD44^+^CD24^−/low^ phenotype of tamoxifen-resistant breast cancer cells and Sox2 expression. Reduction of endogenous Sox2 levels decreases the proportion of the subpopulation of stem/progenitor cells and enhanced Sox2 expression confers tamoxifen resistance to MCF-7 cells *in vitro* and *in vivo*. In addition, Sox2 silencing significantly reversed tamoxifen resistance in both the native (BT474) and another anti-oestrogen resistance model (T47D). Importantly, evidence of the potential clinical relevance was observed in a cohort of ER-positive breast cancer patients who received tamoxifen therapy, where high levels of Sox2 correlated with endocrine treatment failure and poor relapse-free survival. Finally, Sox2 expression leads to Wnt signalling activation and resistance to tamoxifen. Taken together, these findings suggest that Sox2 plays a key role in the development of tamoxifen resistance by maintaining the cells in a more stem cell-like state through increased autocrine Wnt signalling.

Following current anti-cancer treatments a subset of cells, the tumour-initiating cells or cancer stem cells, may reinitiate tumour growth after therapy in many patients. Indeed, radiation-induced enrichment of cancer stem/progenitor cells occurs in MCF-7 breast cancer cells, suggesting that stem/progenitor cells have increased survival mechanisms (Phillips *et al,* 2006; Woodward *et al,* 2007). Further evidence supporting the intrinsic resistance of cancer stem cells to treatment was provided by a study comparing breast cancer core biopsies before and after treatment, which showed that chemotherapy leads to an increase in the proportion of cancer stem cells with the phenotype CD44^+^CD24^−/low^ and to enhanced mammosphere forming efficiency (Li *et al,* 2008). In addition, high ALDH activity identifies cancer stem cells and is associated with poor prognosis (Ginestier *et al,* 2007). Furthermore, ALDH-positive cells are more invasive than the ALDH-negative cell population and have increased metastatic potential (Charafe-Jauffret *et al,* 2009), in agreement with our findings that MCF-7TamR cells are more invasive and show a higher content of ALDH-positive cells.

Breast stem cells have been reported to lack ER or express it at very low levels (Clayton *et al,* 2004), which may facilitate the resistance of cancer stem cells to the antiproliferative effects of tamoxifen. Consistent with this, we show that the cancer stem cells, which express high levels of Sox2, lack or express very low levels of ER and, therefore, they will be more resistant to tamoxifen. In fact, the impact of reducing Sox2 expression on the proportion of stem cells in MCF-7TamR cells suggests that Sox2 plays a relevant role in conferring a less differentiated phenotype. This observation was corroborated in the clinical samples where increased histological grade significantly correlated with Sox2 expression. Accordingly, it was shown that histologically poorly differentiated breast tumours display preferential overexpression of genes normally enriched in ES cells (Ben-Porath *et al,* 2008) and that they contain a higher proportion of cancer stem cells than well-differentiated cancers (Pece *et al,* 2010), supporting the notion that the cancer stem cell content reflects the malignancy of the tumour (Vivanco, 2010).

The mechanisms that contribute to elevated Sox2 levels in resistant cancers are not fully understood. The previously reported differentiating effect of oestrogen on stem cells (Simoes *et al,* 2011) may be partly due to its capacity to repress Sox2, although this effect is similar in parental MCF-7c and MCF-7TamR cells (supplementary Fig 6C), suggesting that high expression of Sox2 in tamoxifen resistant cells cannot simply be explained by lack of ER activity. We observed that oestrogen reduces Sox2 mRNA expression levels already after 4 h, independently of the presence of actinomycin D (supplementary Fig 6D). ER phosphorylation at Serine 118 was found to be increased in MCF-7TamR cells, consistent with previous studies in other resistance models (Chen *et al,* 2013; Sarwar *et al*, 2006). However, changes in Sox2 expression did not affect Serine 118 phosphorylation (supplementary Fig 6E), indicating that the effects of Sox2 do not involve phosphorylation at this site. Interestingly, Sox2 has been proposed to be a possible driver of the basal-like phenotype in sporadic breast cancer because it is expressed frequently in basal-like breast carcinomas (Rodriguez-Pinilla *et al*, 2007). In addition, the level of Sox2 expression is strongly correlated with tumour grade in breast cancer (Chen *et al*, 2008), and high expression of Sox2 has been proposed to increase metastatic potential (Lengerke *et al*, 2011). Furthermore, a 3q copy number gain (that includes the *SOX2* locus) is a stronger predictor of recurrence than grade and other features in invasive breast carcinoma (Janssen *et al*, 2003). Our results indicate that Sox2 is not just implicated in tumourigenesis but is also involved in the development of resistance to therapy. Ectopic expression of Sox2 in MCF-7 cells is sufficient to render them more resistant to tamoxifen treatment *in vitro* and *in vivo*, in association with increases in the frequency of stem cells and capacity for invasion, suggesting a potential mechanism for the development of resistance to endocrine therapy. In addition, our gene expression analysis highlights the differential expression of several genes involved in the response to drugs (supplementary Fig 6B). Consistent with these findings, Sox2 is also implicated in the cancer stem cell phenotype and development of chemoresistance in glioblastoma (Jeon *et al*, 2011) and prostate cancer (Jia *et al*, 2011). Most importantly, our experimental findings were recapitulated in samples derived from breast cancer patients that had received tamoxifen treatment. Intriguingly, increased Sox2 levels also significantly correlated with lower PR expression than in primary tumours, as observed in the resistance models examined, suggesting that the ER signalling pathway is compromised during development of tamoxifen resistance. The use of a larger cohort of patients is now warranted to explore the predictive power of Sox2 for resistance to endocrine therapy.

Wnt signalling has recently been shown to be implicated in the normal physiology of the mammary gland stem cells (Zeng & Nusse, 2010). Furthermore, altered Wnt/β-catenin pathway has been proposed to be implicated in breast tumour initiating cells (Roarty & Rosen, 2010), and a small molecule inhibitor of Wnt secretion was recently reported to halt tumour growth *in vivo* (Proffitt *et al*, 2013). Gene expression profiling of Sox2 overexpressing cells revealed increased expression of the Wnt target genes DKK1 and Axin2. DKK1 has been found preferentially expressed in hormone resistant breast tumours and tumours with poor prognosis (Forget *et al*, 2007). Axin2 can regulate epithelial-mesenchymal transition by controlling Snail1 activity in breast cancer cells (Yook *et al*, 2006) and its expression has been shown to be upregulated in breast tumours (Ayyanan *et al*, 2006). Furthermore, increased Wnt/β-catenin signalling has been shown to be an early event in a model of breast neoplasia (Khalil *et al*, 2012) and to enhance self-renewal and mediate radiation resistance in mammary gland progenitor cells (Chen *et al*, 2007). Our results suggest that Wnt signalling is activated in tamoxifen resistant cells through an autocrine mechanism, since it is blocked by IWP-2, which inhibits Wnt secretion. Moreover, the block can be rescued by exogenous Wnt-3a, which restored hormone resistance to IWP-2-treated tamoxifen resistant cells. Finally, there is reciprocal regulation of Sox2 and Wnt signalling, since not only does Sox2 regulate Wnt activity, but Wnt signals regulate Sox2, as recently reported by Wang and colleagues, who showed that the Lgr4/Wnt/β-catenin/Lef1 pathway controls Sox2 expression (Wang *et al*, 2013). Consistent with these results and with a positive feedback mechanism, we observed that treatment of MCF-7TamR cells with the Wnt inhibitor IWP-2 reduces Sox2 expression (supplementary Fig 6F). Together these observations highlight the relevance of the Wnt/β-catenin signalling pathway in breast cancer and in resistance to tamoxifen.

In normal breast, the expression levels of stem cell markers are downregulated during the differentiation process to epithelial cells, while their expression appears to be “reawakened” in tumour cells (Simoes *et al*, 2011). Significantly, development of tamoxifen resistance implies loss of ER transcriptional activity and elevated Sox2 expression, leading to Wnt signalling activation and enrichment of the cancer stem cell population (supplementary Fig 7). Targeting the Wnt signalling pathway may favour stem cell differentiation and render tumour cells more sensitive to tamoxifen. The implication of these findings is that a combination of tamoxifen and small molecule inhibitors of Wnt signalling could be developed as a new treatment to prevent recurrence in defined groups of breast cancer patients.

## Materials and Methods

### Cell culture and establishment of TamR cells

All cells were obtained from American Type Culture Collection (ATTC). MCF-7 cells were cultured in DMEM:F12 medium with GlutaMAX (Gibco) supplemented with 8% foetal bovine serum, FBS, (Sigma) and 1% penicillin/streptomycin (Sigma) at 37°C in 5% CO_2_. Cells were grown in the presence of ethanol, as vehicle, and 5 × 10^−7^ M 4-OH-tamoxifen (Sigma), respectively, in DMEM:F12 with 8% FBS for 6 months. During this time, the medium was replaced every 3 days and the cell cultures were passaged by trypsinization after 70–80% confluency was reached. During the first few weeks cell growth rates were strongly reduced by tamoxifen treatment (no effect was ever detected by the very small dose (<0.01% v/v) of ethanol provided to the MCF-7 control cells. Eventually, cell growth gradually increased, leading to the development of the tamoxifen resistant cell line MCF-7TamR. These cells were maintained in culture with 4-OH-tamoxifen for a further 4 months before characterization. MCF-7c (parental control) and MCF-7TamR have been routinely maintained in the presence of ethanol, as vehicle, and 5 × 10^−7^ M of 4-OH-tamoxifen, respectively. Their cell growth properties have remained stable since then. BT474 cells were cultured in DMEM:F12 medium with GlutaMAX (Gibco) supplemented with 8% foetal bovine serum, FBS, 5 μg/ml insulin (Sigma) and 1% penicillin/streptomycin (Sigma) at 37°C in 5% CO_2_. MCF-7GFP (MCF-7v) and MCF-7SOX2 overexpressing cells (Simoes *et al*, 2011) were generated by infection with lentivirus encoding GFP (pSin-EF2-EGFP-Pur vector) and Sox2 (pSin-EF2-Sox2-Pur vector), respectively. Mammosphere cultures were maintained as in (Dontu *et al*, 2003). More detailed information can be found in supplementary.

### Proliferation assay

MCF-7, MCF-7c and MCF-7TamR cells were seeded at 10^5^ cells/well in six-well plates in normal medium. Cell numbers were determined by counting with a haemocytometer. The medium was changed after 3 days. MCF-7c and MCF-7TamR cells were seeded in six-well plate at 5 × 10^4^ cells/well in six-well plates and hormone starved in DMEM:F12 containing 8% charcoal-treated FBS for 48 h. Cells were then washed and grown in DMEM:F12 containing 8% charcoal-treated FBS in the presence of 5 × 10^−7^ M tamoxifen or ethanol.

### Mammosphere formation assay

MCF-7c and MCF-7TamR cells were plated in poly-HEMA six-well coated plates at 5000 cells/ml. At day 7, 1 μM of calcein AM (Sigma) was added to each well and incubated for 1 h. After solidification in 0.3% agarose mammospheres bigger than 35 μm diameter were counted using a Metaxpress microscope (Molecular Devices). The mammosphere formation efficiency (shown as percentage) was calculated by dividing the number of mammospheres formed by the original number of single cells seeded.

### Invasion assay

*In vitro* invasion and migration assays were performed as in (Hayashida *et al*, 2010). More detailed information can be found in supplementary.

### Transient transfection and luciferase assay

MCF-7, MCF-7c and MCF-7TamR cells were seeded in six-well plate at 2.5 × 10^5^ cells/well and grown in charcoal-treated conditions for 48 h. The cells were transfected with the ERE-TK-luciferase reporter (kindly provided by Prof. M Parker, London) using Lipofectamine 2000 (Invitrogen) following the manufacturer's instructions. Each well also received pRL β-galactosidase to normalize for transfection efficiency (Vivanco *et al*,^1995^). After transfection, the cells were maintained in phenol red free DMEM:F12 containing 8% charcoal stripped FBS, treated with 10^−8^ M oestrogen or 5 × 10^−7^ M 4-OH-tamoxifen or ethanol (vehicle) for 48 h. The cell lysates were assayed for luciferase and β-galactosidase activities with the Luciferase Assay Kit (Promega) and the Tropix Galacto-light-plus assay (Applied Biosystems), respectively, using a luminometer (Turner Biosystem). The luciferase results are shown as relative light units of luciferase activity normalized with respect to β-galactosidase activity.

### Real-Time Polymerase Chain Reaction (PCR)

RNA was isolated using the TRIzol method (Invitrogen). Real-time PCR was performed on a 7300 Real-Time PCR System (Applied Biosystems). More detailed information can be found in supplementary.

### Western blot

Cell lysates were prepared directly with Laemmli sample buffer (Sigma). Primary antibodies included; mouse anti-ERα (6F11, Novocastra), rabbit anti-phospho ER (S118) (2515, Cell Signaling), mouse anti-PR (Novocastra), goat anti-SOX2 (Y17, Santa Cruz), mouse anti-OCT3/4 (H-134, Santa Cruz), goat anti-NANOG (R&D System), mouse anti-GAPDH (Sigma), anti-β-actin and mouse anti-β-tubulin (Sigma). For detection an enhanced chemiluminescence detection kit (Amersham) was used.

### Immunofluorescence

Cells were cultured on cover slips, fixed with paraformaldehyde (4% for 10 min at 4°C) and permeabilized for 20 min with PBS supplemented with 0.5% of Triton X-100, followed by blocking for 20 min with TBS supplemented with 0.1% of Triton X-100 (Sigma) and 3% of BSA and incubated for 1 h at room temperature with primary antibody: goat anti-SOX2 and mouse anti-ERα, and with secondary antibodies, anti-mouse Alexa 647 (Molecular Probes), anti-mouse Alexa 488 (Molecular Probes), anti-goat Alexa 568 (Molecular Probes) and phalloidin-FITC (Sigma). Slides were mounted in Vectashield with DAPI (Vector). Immunofluorescence of sorted cells was performed on cytospin preparations (800 g for 5 min) as described above. Mammospheres were collected by centrifugation, fixed in paraformaldehyde 4% overnight at 4°C, permeabilized in PBS supplemented with 1% of Triton X-100 for 1 h at room temperature, blocked and incubated overnight at 4°C with primary antibody (anti-SOX2 and anti-ERα) followed by secondary antibodies (anti-mouse Alexa 647, anti-goat Alexa 568) and phalloidin-FITC. Antibody binding was visualized using a Leica confocal microscope. Digital images were processed using Adobe Photoshop CS2 and analysed with ImageJ image-analysis software (W. Rasband, NIH).

### Fluorescence activated cell sorting (FACS)

Human epithelial membrane antigen (EMA) and common acute lymphoblastic leukaemia antigen (CALLA) labelling was performed as previously described (Clayton *et al*, 2004). The mouse PE anti-CD24 antibody (BD, clone ML5) and mouse allophycocyanin (APC) anti-CD44 antibody (BD, clone G44-26) were used to label CD24 and CD44. More detailed information can be found in supplementary.

### ALDEFLUOR assay

The ALDEFLUOR assay was carried out according to manufacturer's (Stemcell Technologies) guidelines. More details provided in supplementary.

### Small interfering and short hairpin RNA transfection

Small interfering RNA oligonucleotides were transfected using Lipofectamine 2000 (Invitrogen) according to the manufacturer's protocol. siRNA oligos (50 nM) were incubated with the cells for 48 h before analysis. To transfect siRNA in suspension culture, 48 h after the first transfection, cells were transfected again with 50 nM of siRNA and allowed to grow in suspension culture for 96 h. The sequences of each Stealth™RNAi (Invitrogen) oligonucleotide are as follows:

siSOX2 1, HSS186041 5′ CCUGUGGUUACCUCUUCCUCCCACU 3′

siSOX2 2, HSS186045 5′ GCGUGAACCAGCGCAUGGACAGUUA 3′.

Two pLKO.1 lentivirus shRNAs vector targeted against SOX2 were purchased from Open Biosystem (sh1: TRCN0000085748; sh2: TRCN0000085750). An empty shRNA vector was used as negative control (shC). Lentiviruses were produced as previously described (Simoes *et al*, 2011).

### Xenograft analysis

All animal procedures were carried out at the SPF animal facility of CIC bioGUNE (AAALAC-accredited) and conducted in accordance with the*Guide for the Care and Use of Laboratory Animals* (Institute of Laboratory Animal Resources NRC, 1996) and with European policies (European Commission, 1986). Protocols were approved by the CIC bioGUNE Bioethical and Animal Welfare Committee. A total of 1 × 10^6^ cells of MCF7c, MCF7TamR or MCF7SOX2 cells were suspended in 100 μl of PBS/Matrigel (1:1) and injected s.c. into female 3- to 4-week-old BALC/c nu/nu athymic mice (Harlan), which simultaneously received a 60-day slow release pellet containing 0.72 mg of 17β-estradiol with or without 5 mg tamoxifen (Innovative Research of America). Animals were observed once a week.

### Cell growth analysis

MTT assays were performed following the manufacturer's instructions. Detailed information for this assay and crystal violet staining can be found in supplementary.

### Immunohistochemistry

Immunohistochemical staining on formalin-fixed, paraffin-embedded carcinomas and non-neoplastic breast tissue was performed using the Leica Bond-III stainer. Sox2 staining was scored on the basis of both the percentage of positive cells and the intensity of the staining according to the Allred score (Harvey *et al*, 1999). Detailed information can be found in supplementary.

### Gene expression microarray analysis

Gene expression profiles were compared between MCF-7v and MCF-7SOX2 cells cultured in adherent or suspension conditions using the Human HT-12 v1 BeadChips (Affymetrix, Santa Clara, CA). Detailed information can be found in supplementary. Microarray data are available in the ArrayExpress database (http://www.ebi.ac.uk/arrayexpress) under accession number E-MEXP-3984.

### Statistical analysis

Data from at least three independent experiments are expressed as means ± SD. Clinical data were analysed as indicated in the figure legends. Each data point of real-time PCR, MTT, mammosphere formation, luciferase activity assays and proliferation was run at least in triplicates and independent experiments were performed at least three times. Student's *t*-test was used to determine statistically significant differences and *p* < 0.05 was considered to be statistically significant unless otherwise specified.

## Author contributions

MP designed and performed experiments, analysed results and prepared the figures; GD and MR contributed to the characterization of the cell lines with modulated Sox2 levels; OI and MR performed the FACS sorting and analyses; BS analysed factors expression in some breast cancer cell lines; VC contributed to the characterization of the resistant cell lines; IB, JALR and IZ managed the collection of human samples; RK conceived and coordinated the Wnt experiments and contributed to the preparation of the manuscript; MV conceived the project, analysed the data and wrote the paper.

## Supplementary Information

for this article is available online: http://embomolmed.embopress.org

## References

[b1] Al-Hajj M, Wicha MS, Benito-Hernandez A, Morrison SJ, Clarke MF (2003). Prospective identification of tumorigenic breast cancer cells. Proc Natl Acad Sci USA.

[b2] Ali S, Coombes RC (2002). Endocrine-responsive breast cancer and strategies for combating resistance. Nat Rev Cancer.

[b3] Ayyanan A, Civenni G, Ciarloni L, Morel C, Mueller N, Lefort K, Mandinova A, Raffoul W, Fiche M, Dotto GP (2006). Increased Wnt signaling triggers oncogenic conversion of human breast epithelial cells by a Notch-dependent mechanism. Proc Natl Acad Sci USA.

[b4] Ben-Porath I, Thomson MW, Carey VJ, Ge R, Bell GW, Regev A, Weinberg RA (2008). An embryonic stem cell-like gene expression signature in poorly differentiated aggressive human tumors. Nat Genet.

[b5] Chanrion M, Negre V, Fontaine H, Salvetat N, Bibeau F, Mac Grogan G, Mauriac L, Katsaros D, Molina F, Theillet C (2008). A gene expression signature that can predict the recurrence of tamoxifen-treated primary breast cancer. Clin Cancer Res.

[b6] Charafe-Jauffret E, Ginestier C, Iovino F, Wicinski J, Cervera N, Finetti P, Hur MH, Diebel ME, Monville F, Dutcher J (2009). Breast cancer cell lines contain functional cancer stem cells with metastatic capacity and a distinct molecular signature. Cancer Res.

[b7] Chen MS, Woodward WA, Behbod F, Peddibhotla S, Alfaro MP, Buchholz TA, Rosen JM (2007). Wnt/beta-catenin mediates radiation resistance of Sca1+ progenitors in an immortalized mammary gland cell line. J Cell Sci.

[b8] Chen Y, Shi L, Zhang L, Li R, Liang J, Yu W, Sun L, Yang X, Wang Y, Zhang Y (2008). The molecular mechanism governing the oncogenic potential of SOX2 in breast cancer. J Biol Chem.

[b9] Chen B, Dodge ME, Tang W, Lu J, Ma Z, Fan CW, Wei S, Hao W, Kilgore J, Williams NS (2009). Small molecule-mediated disruption of Wnt-dependent signaling in tissue regeneration and cancer. Nat Chem Biol.

[b10] Chen M, Cui YK, Huang WH, Man K, Zhang GJ (2013). Phosphorylation of estrogen receptor alpha at serine 118 is correlated with breast cancer resistance to tamoxifen. Oncol Lett.

[b11] Clayton H, Titley I, Vivanco M (2004). Growth and differentiation of progenitor/stem cells derived from the human mammary gland. Exp Cell Res.

[b12] Diehn M, Cho RW, Clarke MF (2009). Therapeutic implications of the cancer stem cell hypothesis. Semin Radiat Oncol.

[b13] Dontu G, Abdallah WM, Foley JM, Jackson KW, Clarke MF, Kawamura MJ, Wicha MS (2003). In vitro propagation and transcriptional profiling of human mammary stem/progenitor cells. Genes Dev.

[b14] Elizalde C, Campa VM, Caro M, Schlangen K, Aransay AM, Vivanco M, Kypta RM (2011). Distinct roles for Wnt-4 and Wnt-11 during retinoic acid-induced neuronal differentiation. Stem Cells.

[b15] Forget MA, Turcotte S, Beauseigle D, Godin-Ethier J, Pelletier S, Martin J, Tanguay S, Lapointe R (2007). The Wnt pathway regulator DKK1 is preferentially expressed in hormone-resistant breast tumours and in some common cancer types. Br J Cancer.

[b16] Ginestier C, Hur MH, Charafe-Jauffret E, Monville F, Dutcher J, Brown M, Jacquemier J, Viens P, Kleer CG, Liu S (2007). ALDH1 is a marker of normal and malignant human mammary stem cells and a predictor of poor clinical outcome. Cell Stem Cell.

[b17] Harvey JM, Clark GM, Osborne CK, Allred DC (1999). Estrogen receptor status by immunohistochemistry is superior to the ligand-binding assay for predicting response to adjuvant endocrine therapy in breast cancer. J Clin Oncol.

[b18] Hayashida T, Takahashi F, Chiba N, Brachtel E, Takahashi M, Godin-Heymann N, Gross KW, Vivanco MM, Wijendran V, Shioda T (2010). HOXB9, a gene overexpressed in breast cancer, promotes tumorigenicity and lung metastasis. Proc Natl Acad Sci USA.

[b19] Janssen EA, Baak JP, Guervos MA, Diest van PJ, Jiwa M, Hermsen MA (2003). In lymph node-negative invasive breast carcinomas, specific chromosomal aberrations are strongly associated with high mitotic activity and predict outcome more accurately than grade, tumour diameter, and oestrogen receptor. J Pathol.

[b20] Jeon HM, Sohn YW, Oh SY, Kim SH, Beck S, Kim S, Kim H (2011). ID4 imparts chemoresistance and cancer stemness to glioma cells by derepressing miR-9*-mediated suppression of SOX2. Cancer Res.

[b21] Jia X, Li X, Xu Y, Zhang S, Mou W, Liu Y, Liu Y, Lv D, Liu CH, Tan X (2011). SOX2 promotes tumorigenesis and increases the anti-apoptotic property of human prostate cancer cell. J Mol Cell Biol.

[b22] Jordan VC, O'Malley BW (2007). Selective estrogen-receptor modulators and antihormonal resistance in breast cancer. J Clin Oncol.

[b23] Khalil S, Tan GA, Giri DD, Zhou XK, Howe LR (2012). Activation status of Wnt/ss-catenin signaling in normal and neoplastic breast tissues: relationship to HER2/neu expression in human and mouse. PLoS ONE.

[b24] Lengerke C, Fehm T, Kurth R, Neubauer H, Scheble V, Muller F, Schneider F, Petersen K, Wallwiener D, Kanz L (2011). Expression of the embryonic stem cell marker SOX2 in early-stage breast carcinoma. BMC Cancer.

[b25] Li X, Lewis MT, Huang J, Gutierrez C, Osborne CK, Wu MF, Hilsenbeck SG, Pavlick A, Zhang X, Chamness GC (2008). Intrinsic resistance of tumorigenic breast cancer cells to chemotherapy. J Natl Cancer Inst.

[b26] Liu S, Ginestier C, Charafe-Jauffret E, Foco H, Kleer CG, Merajver SD, Dontu G, Wicha MS (2008). BRCA1 regulates human mammary stem/progenitor cell fate. Proc Natl Acad Sci USA.

[b27] Osborne CK, Shou J, Massarweh S, Schiff R (2005). Crosstalk between estrogen receptor and growth factor receptor pathways as a cause for endocrine therapy resistance in breast cancer. Clin Cancer Res.

[b28] Pece S, Tosoni D, Confalonieri S, Mazzarol G, Vecchi M, Ronzoni S, Bernard L, Viale G, Pelicci PG, Fiore Di PP (2010). Biological and molecular heterogeneity of breast cancers correlates with their cancer stem cell content. Cell.

[b29] Phillips TM, McBride WH, Pajonk F (2006). The response of CD24^(−/low)^/CD44^+^ breast cancer-initiating cells to radiation. J Natl Cancer Inst.

[b30] Proffitt KD, Madan B, Ke Z, Pendharkar V, Ding L, Lee MA, Hannoush RN, Virshup DM (2013). Pharmacological inhibition of the Wnt acyltransferase PORCN prevents growth of WNT-driven mammary cancer. Cancer Res.

[b31] Reya T, Morrison SJ, Clarke MF, Weissman IL (2001). Stem cells, cancer, and cancer stem cells. Nature.

[b32] Roarty K, Rosen JM (2010). Wnt and mammary stem cells: hormones cannot fly wingless. Curr Opin Pharmacol.

[b33] Rodriguez-Pinilla SM, Sarrio D, Moreno-Bueno G, Rodriguez-Gil Y, Martinez MA, Hernandez L, Hardisson D, Reis-Filho JS, Palacios J (2007). Sox2: a possible driver of the basal-like phenotype in sporadic breast cancer. Mod Pathol.

[b34] Sarwar N, Kim JS, Jiang J, Peston D, Sinnett HD, Madden P, Gee JM, Nicholson RI, Lykkesfeldt AE, Shousha S (2006). Phosphorylation of ERalpha at serine 118 in primary breast cancer and in tamoxifen-resistant tumours is indicative of a complex role for ERalpha phosphorylation in breast cancer progression. Endocr Relat Cancer.

[b35] Sheridan C, Kishimoto H, Fuchs RK, Mehrotra S, Bhat-Nakshatri P, Turner CH, Goulet R, Badve S, Nakshatri H (2006). CD44^+^/CD24^−^breast cancer cells exhibit enhanced invasive properties: an early step necessary for metastasis. Breast Cancer Res.

[b36] Simoes BM, Piva M, Iriondo O, Comaills V, Lopez-Ruiz JA, Zabalza I, Mieza JA, Acinas O, Vivanco MD (2011). Effects of estrogen on the proportion of stem cells in the breast. Breast Cancer Res Treat.

[b37] Vivanco M (2010). Function follows form: defining mammary stem cells. Sci Transl Med.

[b38] Vivanco MD, Johnson R, Galante PE, Hanahan D, Yamamoto KR (1995). A transition in transcriptional activation by the glucocorticoid and retinoic acid receptors at the tumor stage of dermal fibrosarcoma development. EMBO J.

[b39] Wang Y, Dong J, Li D, Lai L, Siwko S, Li Y, Liu M (2013). Lgr4 regulates mammary gland development and stem cell activity through the pluripotency transcription factor Sox2. Stem Cells.

[b40] Woodward WA, Chen MS, Behbod F, Alfaro MP, Buchholz TA, Rosen JM (2007). WNT/beta-catenin mediates radiation resistance of mouse mammary progenitor cells. Proc Natl Acad Sci USA.

[b41] Yook JI, Li XY, Ota I, Hu C, Kim HS, Kim NH, Cha SY, Ryu JK, Choi YJ, Kim J (2006). A Wnt-Axin2-GSK3beta cascade regulates Snail1 activity in breast cancer cells. Nat Cell Biol.

[b42] Zeng YA, Nusse R (2010). Wnt proteins are self-renewal factors for mammary stem cells and promote their long-term expansion in culture. Cell Stem Cell.

