# Supplementary material for: Sox2 promotes tamoxifen resistance in breast cancer cells
Source: EMBO Mol Med. 2013 Oct 31;6(1):66–79. doi: 10.1002/emmm.201303411 (PMC3936493; doi:10.1002/emmm.201303411)
Supplement: Supplementary file 1 [file emmm0006-0066-sd1.pdf]

# Sox2 promotes tamoxifen resistance in breast cancer cells

Marco Piva, Giacomo Domenici, Oihana Iriondo, Miriam Rábano, Bruno Simões, Valentine Comaills, Inmaculadda Barredo, Jose A. López-Ruiz, Ignacio Zabalza, Robert Kypta and Maria dM. Vivanco

*Corresponding author: Maria dM. Vivanco, CIC bioGUNE*

**Review timeline:**

| | |
|---|---|
| Submission date: | 18 December 2012 |
| Editorial Decision: | 25 January 2013 |
| Resubmission: | 20 August 2013 |
| Editorial Decision: | 16 September 2013 |
| Revision received: | 20 September 2013 |

## Transaction Report:

(Note: With the exception of the correction of typographical or spelling errors that could be a source of ambiguity, letters and reports are not edited. The original formatting of letters and referee reports may not be reflected in this compilation.)

*Editors: Natascha Bushati and Roberto Buccione*

1st Editorial Decision 25 January 2013

Thank you for the submission of your manuscript "Sox2 promotes tamoxifen resistance in breast cancer cells", which has been assigned to me as the handling editor. We have now heard back from the two referees whom we asked to evaluate your manuscript.

As you will see, the referees acknowledge that the overall topic of the manuscript is potentially interesting. However, they also raise significant concerns, that I am afraid preclude publication of the manuscript in EMBO Molecular Medicine.

Both reviewers highlight significant experimental limitations. Most importantly, the reviewers would require key experiments to be repeated in additional tamoxifen-resistant breast cancer cell lines. In addition, reviewer 1 raises important criticisms in terms of the clinical relevance of the results. Specifically, this reviewer would require the analysis of Sox2 expression in a larger cohort of patient-derived primary tumors to substantiate the clinical impact of the study.

Given the concerns raised, I unfortunately see no other choice, but to return your manuscript with the message that we cannot offer to publish it.

I am sorry to disappoint you on this occasion.

***** Reviewer's comments *****

Referee #1 (Remarks):

This manuscript explores the role of Sox2 in tamoxifen resistance. The authors generate a tamoxifen resistant MCF7 derivative and show that Sox2 is elevated. They show that the resistant cell line has

a higher proportion of progenitor cells and that Sox2 is important for growth of the Tam-R cells. They show that Sox2 expression is higher in tumors that relapse and they implicate the Wnt pathway in Sox2 function.

This is an interesting manuscript on an important area of work. That said, some of the work is very descriptive and almost all of the work hinges on a single resistant cell line clone.

- This paper would benefit from a few validation experiments in another resistant cell line, even for some of the basic endpoints (i.e. Sox2 expression, dependence on Sox2). There are many resistant cell lines in the literature and they have very different characteristics. As such, the Tam-R cell line used here may not represent other models.
- The Sox2 overexpression in the xenografts is convincing, but I'm not overly convinced by the tumor data. What if Sox2 is simply an ER repressed gene and elevated levels are a result of lack of ER function? Can IHC of Sox2 in a larger cohort of primary tumors be done and would Sox2 function as a predictive factor in the presence of tamoxifen?

Referee #2 (Comments on Novelty/Model System):

The model system proposed in the current study was inadequate because the authors focused exclusively on MCF-7 cells. It is well known that the MCF-7 cell line varies from lab to lab and that the development of tamoxifen resistance in these cells may differ from other ER-positive breast cancer cells. Therefore, it would strengthen the paper if the authors were to use additional tamoxifen-resistant breast cancer cell lines.

Referee #2 (Remarks):

Manuscript Summary: Hormonal therapies such tamoxifen and aromatase inhibitors are widely used to treat estrogen receptor (ER)-positive breast cancer; however, the majority of patients will eventually develop resistance to treatment. Acquired resistance to endocrine therapy thus represents a serious clinical problem for patients. Recent findings suggest that tumors contain a subpopulation of cancer cells that share some properties with stem/progenitor cells. These tumor-initiating or cancer stem cells are relatively resistant to ionizing radiation and chemotherapy and there is evidence that these cells might potentially play a role in the development of tamoxifen resistance in breast cancer. In the present study, the authors investigated the role of the embryonic stem cell regulator Sox2 [SRY (sex determining region Y)-box 2] in the development of tamoxifen resistance in breast cancer. Sox2 is a transcription factor that is essential for maintaining the survival and pluripotency of undifferentiated embryonic stem cells, and has an emerging role as an epigenetic reprogramming factor and oncogene. In human embryonic stem cells Sox2 regulates the expression of many genes including stem cell genes Oct4 and Nanog. The authors reported that, compared with tamoxifen-sensitive breast cancer cells (MCF-7), tamoxifen resistant (TamR) cells were more invasive and showed increased stem/progenitor cell properties, as measured using mammosphere formation assays and that Sox2 was highly expressed in the TamR cells and in recurrent tumors from patients who had been initially responded to tamoxifen but then failed. In addition, the authors reported that overexpression of Sox2 was sufficient to render cells resistant to tamoxifen in vitro and in vivo. Gene expression profiling highlighted the activation of Wnt signalling in Sox2 overexpressing cells and siRNA knockdown experiment indicated that the Wnt signalling pathway was important for sensitizing the resistant cells to tamoxifen. The authors concluded from their findings that the development of tamoxifen resistance is driven by Sox2-dependent activation of Wnt signalling in cancer stem/progenitor cells.

Questions that need to be addressed:

1) Why did the authors choose to use only MCF-7 breast cancer cells? Do other ER-positive breast cancer cell lines that are resistant to tamoxifen also shown enhanced Sox2 expression? BT474 cells are known to overexpress HER-2 and these cells are resistant to tamoxifen. Have the authors examined these cells to see whether Sox2 plays a role in the development of tamoxifen resistance in these cells? How about cells that are resistant to aromatase inhibitors (AIs)? Do AI-resistant cells

also exhibit enhanced Sox2 expression? The findings with MCF-7 cells might be unique to this particular cell line as oppose to a universal phenomenon. The authors need to clarify this point.
2) The authors mentioned that Sox2 expression is inversely correlated with ERa expression in the TAM-resistant cells. Could the loss of tamoxifen sensitivity in the tamoxifen-resistant MCF-7 cells be due to a loss of ERa expression and/or function rather than overexpression of Sox2? Also, why is progesterone receptor expression suppressed in the tamoxifen-resistant cells? Is that an indication that the ER is no longer functional? If the ER is no longer functional or is partially functional (as indicated by the ERE luciferase assay) then it would be expected that the tamoxifen-resistant cells would not respond to tamoxifen at the same level as the parental MCF-7 cells which express high levels of PgR. This point needs to be addressed. Also, did the authors measure ERa phosphorylation status in the tamoxifen-resistant cells versus the parental cells? Enhanced ERa phosphorylation is known to play a role in tamoxifen resistance hence it is important that the authors examine whether ERa phosphorylation status in altered in the Sox2 overexpressing cells and whether suppression of Sox2 alters ERa phosphorylation.

3) Since siRNA knockdown of Sox2 is able to reverse the tamoxifen-resistant phenotype in TAM-resistant cells....how about overexpression of Sox2 in MCF-7 cells? Does Sox2 overexpression in MCF-7 cells cause these cells to become resistant to tamoxifen? Also, does suppression of Sox2 cause the resistant cells to undergo apoptosis with tamoxifen or is the inhibitory effect of tamoxifen due to cell cycle arrest?

4) How is Sox2 regulated in tamoxifen-resistant versus parental MCF-7 cells and what role if any does ERa play in the process? Does the Sox2 promoter contain an ERE binding site?

5) What is the mechanism by which Sox-2/Wnt signaling pathway contribute to the development of tamoxifen resistance?

6) Is the "stemness" of tamoxifen-resistant cells due primarily to SOX2 overexpression? What role if any do the other stem cell gene regulators NANOG and OCT4 play in the process? NANOG and OCT4 mRNA levels were significantly higher than SOX2 mRNA level in parental MCF-7 cells versus the TAM-R cells. Why is that? This point should be clearly addressed in the discussion.

Resubmission 20 August 2013

We hereby submit a new revised version of the manuscript "Sox2 promotes tamoxifen resistance in breast cancer cells" (EMM-2012-02359-V2-Q). At that time of the original submission, given the concerns raised by the reviewers, you were not able to accept the manuscript. We felt that the concerns raised were appropriate and that by addressing them we would very much improve the quality of the manuscript. Therefore, we have done just that: answered all the points raised, as indicated below. This new manuscript includes all the experiments requested by the reviewers and an extended discussion. We would very much appreciate it if now you would consider this manuscript for publication in *EMBO Molecular Medicine*.

Referee #1:

1. This referee appreciated "the interest of the manuscript and its important area of work", but argued "this paper would benefit from a few validation experiments in another resistant cell line, even for some of the basic endpoints (i.e. Sox2 expression, dependence on Sox2)".

- Whether Sox2 overexpression is a molecular feature shared by other anti-estrogen resistance models is an interesting question. We have now carried out validation experiments using tamoxifen-resistant T47D cells (new Supporting Information Fig 4C and 4D) and also using BT-474 cells (new Fig 5G and Supporting Information Fig 3H) (as suggested by Referee #2). We show that upon development of resistance to tamoxifen, Sox2 expression levels are higher than in parental cells. Moreover, specific down-regulation of Sox2 renders the cells more sensitive to the anti-proliferative

effects of tamoxifen. These findings further support the general importance of Sox2 in the development of resistance to tamoxifen in ER-positive breast cancer cells.

*2. "What if Sox2 is simply an ER repressed gene and elevated levels are a result of lack of ER function? Can IHC of Sox2 in a larger cohort of primary tumors be done and would Sox2 function as a predictive factor in the presence of tamoxifen?*

- To address this point we have taken two parallel and complimentary approaches. On the one hand we have increased the number of cases in the cohort of primary tumours (n = 55, instead of 26 in the earlier manuscript) (new Fig 6). Most importantly, the preliminary data obtained previously are fully supported by this extended analysis. Sox2 is consistently more highly expressed in primary tumours that are not responsive to tamoxifen, while the responsive tumours present a very low level of expression of Sox2.

- On the other hand, we have explored the possibility suggested by the reviewer that Sox2 could function as a predictive factor in the presence of tamoxifen. To this end, we performed *in silico* analysis of 3 independent data sets of ER-positive breast cancer patients, who received tamoxifen therapy, with high and low Sox2 levels. This analysis also suggested a role for Sox2 as prognostic factor for both overall survival and recurrence-free survival (new Fig 6 and Supporting Information Fig 5).

Referee #2:

*1. Why did the authors choose to use only MCF-7 breast cancer cells? Do other ER-positive breast cancer cell lines that are resistant to tamoxifen also shown enhanced Sox2 expression? BT474 cells are known to overexpress HER-2 and these cells are resistant to tamoxifen. Have the authors examined these cells to see whether Sox2 plays a role in the development of tamoxifen resistance in these cells? How about cells that are resistant to aromatase inhibitors (AIs)? Do AI-resistant cells also exhibit enhanced Sox2 expression? The findings with MCF-7 cells might be unique to this particular cell line as oppose to a universal phenomenon. The authors need to clarify this point.*

- This link between Sox2 and tamoxifen resistance in other tumour cell lines is an important point raised also by Referee #1 that has now been addressed (see above, point 1). We have not claimed that Sox2 is implicated in resistance to aromatase inhibitors (AIs). Breast tumours have been shown to use different strategies to adapt to the hormone deprivation caused by treatment with AIs and development of resistance is very common. Multiple reports have indicated the role of pro-growth or apoptotic signalling pathways in the acquisition of resistance to AIs. However, it is interesting that an expression array analysis comparing parental MCF7 cells with cells that have acquired resistance to estrogen deprivation included data suggesting that resistant cells overexpress Sox2, however, although this observation was reported it was not tested further (Lewis-Wambi et al., Eur J Cancer 2008, 44, 1770). As mentioned above, we have now examined the role of Sox2 in several tamoxifen-resistant cells and therefore we can conclude that this is not unique to MCF7 cells.

*2. The authors mentioned that Sox2 expression is inversely correlated with ERa expression in the TAM-resistant cells. Could the loss of tamoxifen sensitivity in the tamoxifen-resistant MCF-7 cells be due to a loss of ERa expression and/or function rather than overexpression of Sox2? Also, why is progesterone receptor expression suppressed in the tamoxifen-resistant cells? Is that an indication that the ER is no longer functional? If the ER is no longer functional or is partially functional (as indicated by the ERE luciferase assay) then it would be expected that the tamoxifen-resistant cells would not respond to tamoxifen at the same level as the parental MCF-7 cells which express high levels of PgR. This point needs to be addressed. Also, did the authors measure ERa phosphorylation status in the tamoxifen-resistant cells versus the parental cells? Enhanced ERa phosphorylation is known to play a role in tamoxifen resistance hence it is important that the authors examine whether ERa phosphorylation status in altered in the Sox2 overexpressing cells and whether suppression of Sox2 alters ERa phosphorylation.*

- The reviewer wonders whether the loss of sensitivity to tamoxifen is due to a loss of ERalpha expression and/or function rather than overexpression of Sox2. We feel it is unlikely that tamoxifen resistance results from loss of ER expression since MCF7TamR cells express similar levels of ER protein as parental cells (Fig 1D). Moreover, gene silencing of Sox2 in MCF7TamR cells (Fig 5F) and ectopic expression of Sox2 in MCF7 cells (Fig 5D) affects tamoxifen sensitivity without significantly affecting ER expression levels (new Supporting Information Fig 6E). However, ER function is clearly altered in MCF7TamR cells, as revealed by reduced ERE-dependent gene reporter activity (Fig 1E) and reduced expression of its target, PR (Fig 1F), and we agree that the changes may be linked to the loss of sensitivity to tamoxifen (Fig 1B). We have now addressed this point more clearly in the discussion.

- Regarding the ER phosphorylation status, previous reports have shown the relevance of serine 118 in tamoxifen resistant tumours. We have found that the steady-state levels of P-Ser118 are elevated in TamR cells (new Supporting Information Fig 6E). This is in agreement with published reports in other tamoxifen resistant models (for example, Chen et al., 2013 and Sarwar et al., 2006). However, we did not observe changes in Ser118 phosphorylation in response to ectopic expression of Sox2 in MCF7 cells or Sox2 downregulation in TamR cells (new Supporting Information Fig 6E).

*3) Since siRNA knockdown of Sox2 is able to reverse the tamoxifen-resistant phenotype in TAM-resistant cells....how about overexpression of Sox2 in MCF-7 cells? Does Sox2 overexpression in MCF-7 cells cause these cells to become resistant to tamoxifen? Also, does suppression of Sox2 cause the resistant cells to undergo apoptosis with tamoxifen or is the inhibitory effect of tamoxifen due to cell cycle arrest?*

- Yes, we already addressed the first question in the previous manuscript, where we showed that overexpression of Sox2 in MCF7 cells is sufficient to render cells more resistant to tamoxifen (Fig 5D), more tumorigenic *in vivo* (Fig 5E) and with increased Wnt signalling activity (Fig 7A and Fig 7C) (and with increased CSC content in the cancer cell population, Simoes et al., 2011), compared to control cells.

- We have now carried out BrdU incorporation assays and find no significant differences in the response to tamoxifen between cells expressing different levels of Sox2 (new Supporting Information Fig 4A). In addition, we have examined the levels of apoptosis using Annexin V and found a significant increase in apoptosis in response to tamoxifen in cells with reduced Sox2 expression levels (new Supporting Information Fig 4B).

*4. How is Sox2 regulated in tamoxifen-resistant versus parental MCF-7 cells and what role if any does ERa play in the process? Does the Sox2 promoter contain an ERE binding site?*

- Several groups have reported genome-wide analysis of ER binding sites, mainly using ChIP-on-chip, and Sox2 was not found to be a target in these lists. However, we have found that Sox2 expression levels are reduced by estrogen treatment at early time points (4 hours), suggesting a direct effect (actinomycin D treatment) (new Supporting Information Fig 6C). In addition, it has been recently reported that Sox2 expression is controlled by the Lgr4/Wnt/beta-catenin/Lef1 pathway (Wang et al., Stem Cells, 2013, May 27). Indeed, we have also observed that inhibition of Wnt signalling using IWP2 results in Sox2 reduction (new Supporting Information Fig 6D). These results suggest that Sox2 regulation is rather complex, with at least two possible mechanisms by which Sox2 is regulated during development of resistance to tamoxifen.

*5. What is the mechanism by which Sox-2/Wnt signaling pathway contribute to the development of tamoxifen resistance?*

- As discussed in the point above, Sox2 induces canonical Wnt signaling pathway, others have recently reported that Lgr4/Wnt/beta-catenin/Lef1 controls Sox2 expression and we have observed that inhibiting Wnt signalling reduces Sox2 levels (new Supporting Information Fig 6D). Together, these findings suggest a positive feed-back mechanism that may contribute to tamoxifen resistance. This potential mechanistic explanation has now been incorporated into the discussion.

*6. Is the "stemness" of tamoxifen-resistant cells due primarily to SOX2 overexpression? What role if any do the other stem cell gene regulators NANOG and OCT4 play in the process? NANOG and OCT4 mRNA levels were significantly higher than SOX2 mRNA level in parental MCF-7 cells versus the TAM-R cells. Why is that? This point should be clearly addressed in the discussion.*

- We agree with the reviewer that this point should have been better clarified. In the previous manuscript we showed that Sox2 is particularly increased (approximately 30-fold at the level of mRNA) during development of tamoxifen resistance, while Nanog is mildly increased (approximately 1.5-fold) and Oct4 appeared slightly reduced (Fig 2A). Furthermore, we showed that Sox2 expression levels are also significantly increased during mammosphere formation (Fig 3B), in contrast, although the expression of Nanog and Oct4 was higher in mammospheres than in adherent cultures, as also previously shown by our lab (Simoes et al., 2011), there were no significant differences between Nanog and Oct4 expression levels in MCF7TamR and control cells (Supporting Information Fig 1A). Finally, and most important, we find that overexpression of Oct4 or Nanog in MCF7 cells is not sufficient to induce resistance to tamoxifen (new Supporting Information Fig 3F), in contrast to the results observed with Sox2 (Fig 5D). This point now is more clearly addressed in the discussion.

We would like to thank the reviewers because we believe that after addressing their points, the manuscript is now very much improved. We do hope that you agree and therefore you will be willing to consider this manuscript for publication in *EMM*.

## 2nd Editorial Decision 16 September 2013

Thank you for the submission of the revised version manuscript EMM-2013-03411 (formerly EMM-2013-02359) to EMBO Molecular Medicine.

We have now received the enclosed reports from the Reviewers that were asked to assess it. As you will see the Reviewers are now supportive and I am pleased to inform you that we will be able to accept your manuscript pending the following final amendments:

1) Reviewer 1 has one remaining point concerning whether Sox2 is an ER repressed gene. Although I will not be asking your to provide further experimental support at this time, I do invite you to address this issue in the manuscript.

2) As per our Author Guidelines, the description of all reported data that includes statistical testing must state the name of the statistical test used to generate error bars and P values, the number (n) of independent experiments underlying each data point (not replicate measures of one sample), and the actual P value for each test (not merely 'significant' or 'P < 0.05').

Please submit your revised manuscript within two weeks. I look forward to seeing a revised form of your manuscript as soon as possible.

***** Reviewer's comments *****

Referee #1 (Remarks):

The authors have addressed most of my concerns. However, my point was whether Sox2 was an ER repressed gene and whether this was simply reflective of changes in ER repression (i.e when estrogen is added to wild type cells, does Sox2 levels go down? And in Tam-R cells is Sox2 high simply because ER isn't repressing it anymore). This was not addressed. That said, the new data adds to the manuscript and it is worthy of publication.

Referee #2 (Comments on Novelty/Model System):

The referees of the former manuscript have raised a lot of comments and questions and asked for a lot of additional experiments. To my opinion, these questions have been adressed well and the additional experiments have improved the manuscript considerably.

Referee #3 (Comments on Novelty/Model System):

The present mauscript entitled "Sox2 promotes tamoxifen resistance in breast cancer cells" by Piva et al. was previously reviewed and was found to be lacking in several key areas. Since the original submission, the authors have made substantial changes/revisions to the manscript to address all of the concerns previously raised. In light of these changes I believe that the manuscript is now acceptable for publication in EMBO Molecular Medicine.

Referee #3 (Remarks):

Endocrine resistance, whether de novo or acquired, remains a
key challenge in the treatment of hormone positive breast cancer.
Mechanisms for resistance to tamoxifen or aromatse inhibitors are varied with evidence existing for genomic and non-genomic cross talk between estrogen receptor ER) and key intracellular signaling pathways. The current study has identified Sox2 as a key promoter of tamoxifen resistance in breast cancer and it suggests that the Wnt signaling pathway is involved in regulating the resistant phenotype. I think the authors have provided strong lab as well as clinical evidence to support the involvement of Sox2 in tamoxifen resistance and it suggest that it might be an important target to reverse resistance.

Revision - authors' response 20 September 2013

Please find enclosed the revised version of the manuscript "Sox2 promotes tamoxifen resistance in breast cancer cells" (EMM-2013-03411). This version now includes the requested final amendments.

Amendment #1:

Reviewer 1 had one remaining point concerning whether Sox2 is an ER repressed gene. You did not request further experimental support, but suggested we address this issue in the manuscript.

- We had already performed these experiments previously and therefore the results have been included (as Supporting Information Fig 6C). We observed that Sox2 is repressed by estrogen in both parental and resistant cells to a similar extent and therefore unlikely to explain the high expression of Sox2 in tamoxifen resistant cells. This issue is addressed in the discussion (page 14).

Amendment #2:

The description of the statistical information needed to be completed.

- Following the Author Guidelines we have included the name of the statistical test used, the number of independent experiments performed and the actual *p* value for each test.

Once again we would like to thank the reviewers because addressing the points they raised has considerably improved the manuscript. We also would like to thank you for considering this manuscript for publication in *EMBO Molecular Medicine* and do hope that with the described amendments you will be able to accept the manuscript.
