# Supplementary material for: Sox2 promotes tamoxifen resistance in breast cancer cells
Source: EMBO Mol Med. 2013 Oct 31;6(1):66–79. doi: 10.1002/emmm.201303411 (PMC3936493; doi:10.1002/emmm.201303411)
Supplement: Supplementary file 2 [file emmm0006-0066-sd2.pdf]

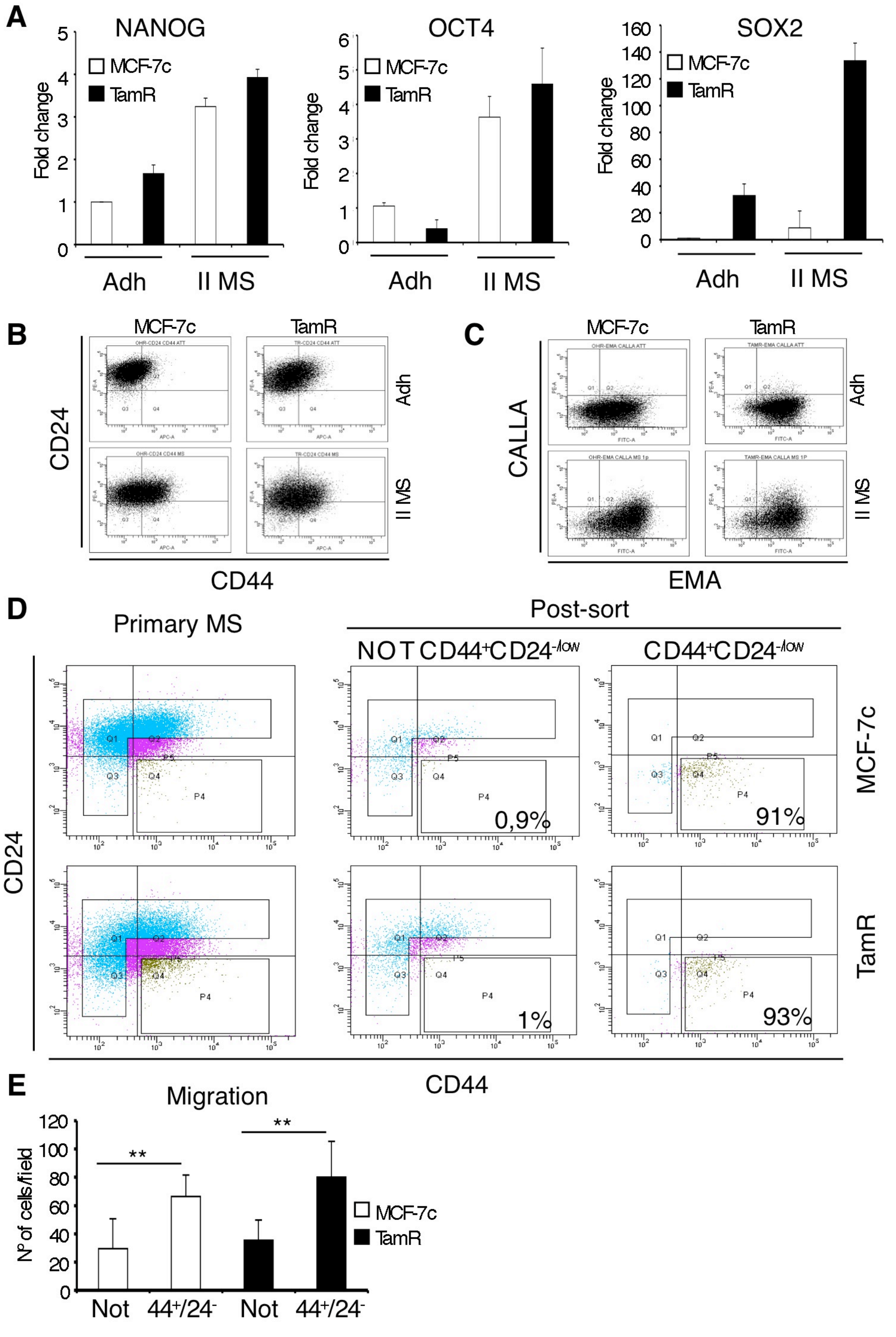
A
NANOG
OCT4
SOX2
Fold change
MCF-7c
TamR
Adh
II MS
B
C
MCF-7c
TamR
CD24
CD44
CALLA
EMA
Adh
II MS
D
Primary MS
Post-sort
NOT CD44+CD24-/low
CD44+CD24-/low
0,9%
91%
1%
93%
MCF-7c
TamR
CD24
CD44
E
Migration
Nº of cells/field
**
MCF-7c
TamR
Not
44+/24-

Sup. Figure 1. Characterisation of MCF-7TamR cells.

A. Transcript levels of NANOG, OCT4 and SOX2 in MCF-7c and MCF-7TamR (TamR) cells grown as adherent cells (Adh) or as secondary mammospheres (II MS) were quantified by real-time PCR and presented as fold induction with MCF-7c value set as 1 (n = 3). **B-C**. Plots of one representative experiment of flow cytometry analysis of the **B**. $CD44^{+}/CD24^{-/low}$ and **C.** $EMA^{+}/CALLA^{+}$ stem cell populations in MCF-7c and MCF-7TamR cells grown as adherent cells (Adh) or as secondary mammospheres (II MS). **D.** Representative example of flow cytometry analysis of the $CD44^{+}/CD24^{-/low}$ stem cell populations in MCF-7c and MCF-7TamR cells grown as primary mammospheres showing the gates to separate the $CD44^{+}/CD24^{-/low}$ and NOT population (left) and the efficiency of the sorting (centre and right). **E**. *In vitro* migration capacity of MCF7c and MCF-7TamR secondary mammospheres FACS sorted for CD24 and CD44 (n = 4) $^{**}p = 0.001$ by t-test.
