# Supplementary material for: Sox2 promotes tamoxifen resistance in breast cancer cells
Source: EMBO Mol Med. 2013 Oct 31;6(1):66–79. doi: 10.1002/emmm.201303411 (PMC3936493; doi:10.1002/emmm.201303411)
Supplement: Supplementary file 3 [file emmm0006-0066-sd3.pdf]

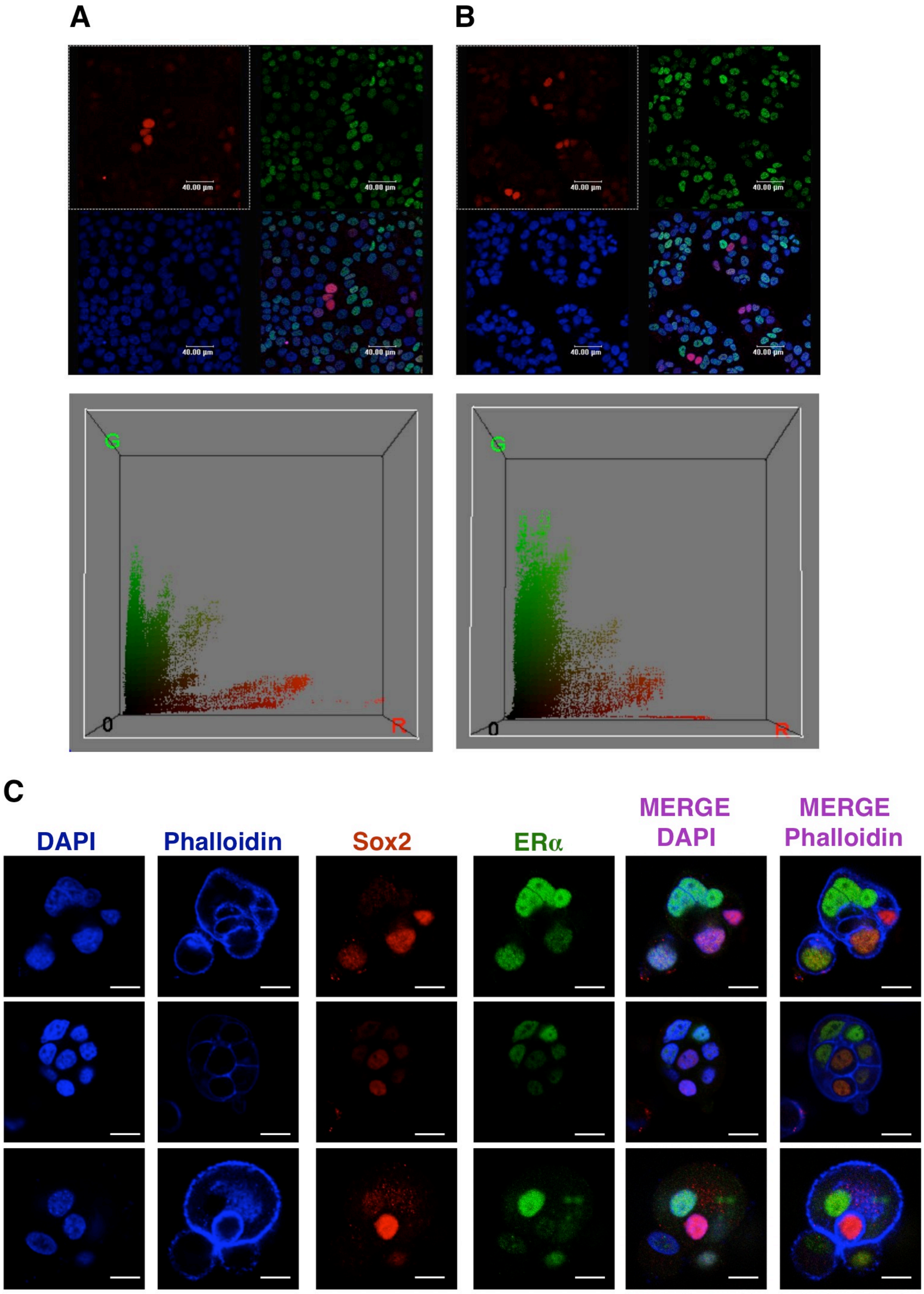

Sup. Figure 2. Sox2 and ER expression.

Sox2 and ER expression was visualised by immunofluorescence in **A.** T-47D and **B.** ZR-75-1 cells grown in adherent conditions (Bar = 40 μm). **C.** Expression of Sox2 and ER was visualised by immunofluorescence in MCF-7TamR cells grown as secondary mammospheres (Bar = 10 μm).
