# Supplementary material for: Sox2 promotes tamoxifen resistance in breast cancer cells
Source: EMBO Mol Med. 2013 Oct 31;6(1):66–79. doi: 10.1002/emmm.201303411 (PMC3936493; doi:10.1002/emmm.201303411)
Supplement: Supplementary file 4 [file emmm0006-0066-sd4.pdf]

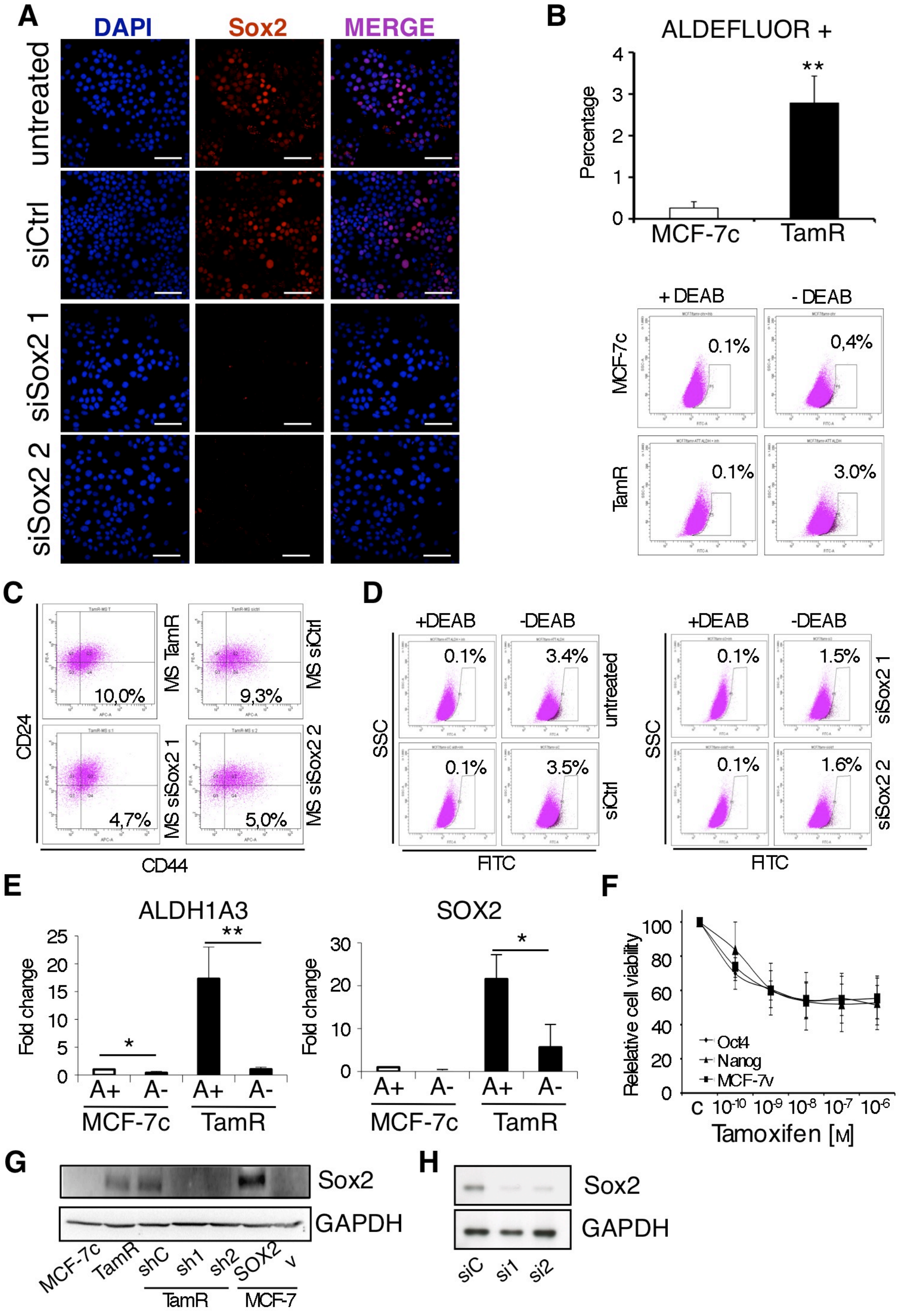
A
DAPI
Sox2
MERGE
untreated
siCtrl
siSox2 1
siSox2 2
B
ALDEFLUOR +
Percentage
**
MCF-7c
TamR
+ DEAB
- DEAB
0.1%
0,4%
3.0%
C
CD24
CD44
MS TamR
MS siCtrl
MS siSox2 1
MS siSox2 2
10,0%
9,3%
4,7%
5,0%
D
+DEAB
-DEAB
SSC
FITC
untreated
siCtrl
siSox2 1
siSox2 2
0.1%
3.4%
3.5%
1.5%
1.6%
E
ALDH1A3
SOX2
Fold change
A+
A-
F
Relative cell viability
Oct4
Nanog
MCF-7v
Tamoxifen [M]
G
Sox2
GAPDH
MCF-7c
TamR
shC
sh1
sh2
SOX2
v
MCF-7
H
siC
si1
si2

Sup. Figure 3. Modulation of Sox2 expression levels.

A. Immunofluorescence analysis of Sox2 expression levels in adherent MCF-7TamR cells transfected with 2 different Sox2 siRNA sequences (siSox2 1 and 2) as well as a control siRNA sequence (siCtrl) (Bar = 50 μm). **B.** ALDEFLUOR positive population in adherent MCF-7c and MCF-7TamR cells (n = 3) **p = 0.002 by t-test. One representative FACS analysis is shown. **C.** Representative example of flow cytometry analysis of the $CD44^{+}/CD24^{-/low}$ stem cell populations in MCF-7TamR cells transfected with 2 different Sox2 siRNA sequences (siSox2 1 and 2) as well as a control siRNA sequence (siCtrl) grown as mammospheres. **D.** Representative example of flow cytometry analysis of ALDEFLUOR population in MCF-7TamR cells transfected with 2 different Sox2 siRNA sequences (siSox2 1 and 2) as well as a control siRNA sequence (siCtrl). **E.** ALDH1A3 (**p = 0.002 by t-test) and SOX2 (*p = 0.04 by t-test) mRNA expression levels in ALDEFLUOR positive (A+) and negative (A-) population of MCF-7c and MCF-7TamR (TamR) cells quantified by real-time PCR (n = 4). **F**. Proliferation analysis of control MCF-7v (v) cells and MCF-7 cells overexpressing Nanog (Nanog) and Oct4 (Oct4) growing in presence of tamoxifen by crystal violet (n = 4). **G.** Western blot analysis of Sox2 and GAPDH (loading control) expression levels in adherent MCF7c, MCF-7TamR cells, MCF-7TamR cells stably transfected with 2 different Sox2 shRNA sequences (sh1 and sh2) as well as a control shRNA sequence (shC) and MCF-7 cells stably overexpressing Sox2 (Sox2) and control MCF-7v cells (v). **H.** Western blot analysis of Sox2 levels and GAPDH (loading control) in BT474 cells transfected with two specific Sox2 siRNA sequences (si1, si2, as in A) and control siRNA sequence (siC).
