# Supplementary material for: Sox2 promotes tamoxifen resistance in breast cancer cells
Source: EMBO Mol Med. 2013 Oct 31;6(1):66–79. doi: 10.1002/emmm.201303411 (PMC3936493; doi:10.1002/emmm.201303411)
Supplement: Supplementary file 5 [file emmm0006-0066-sd5.pdf]

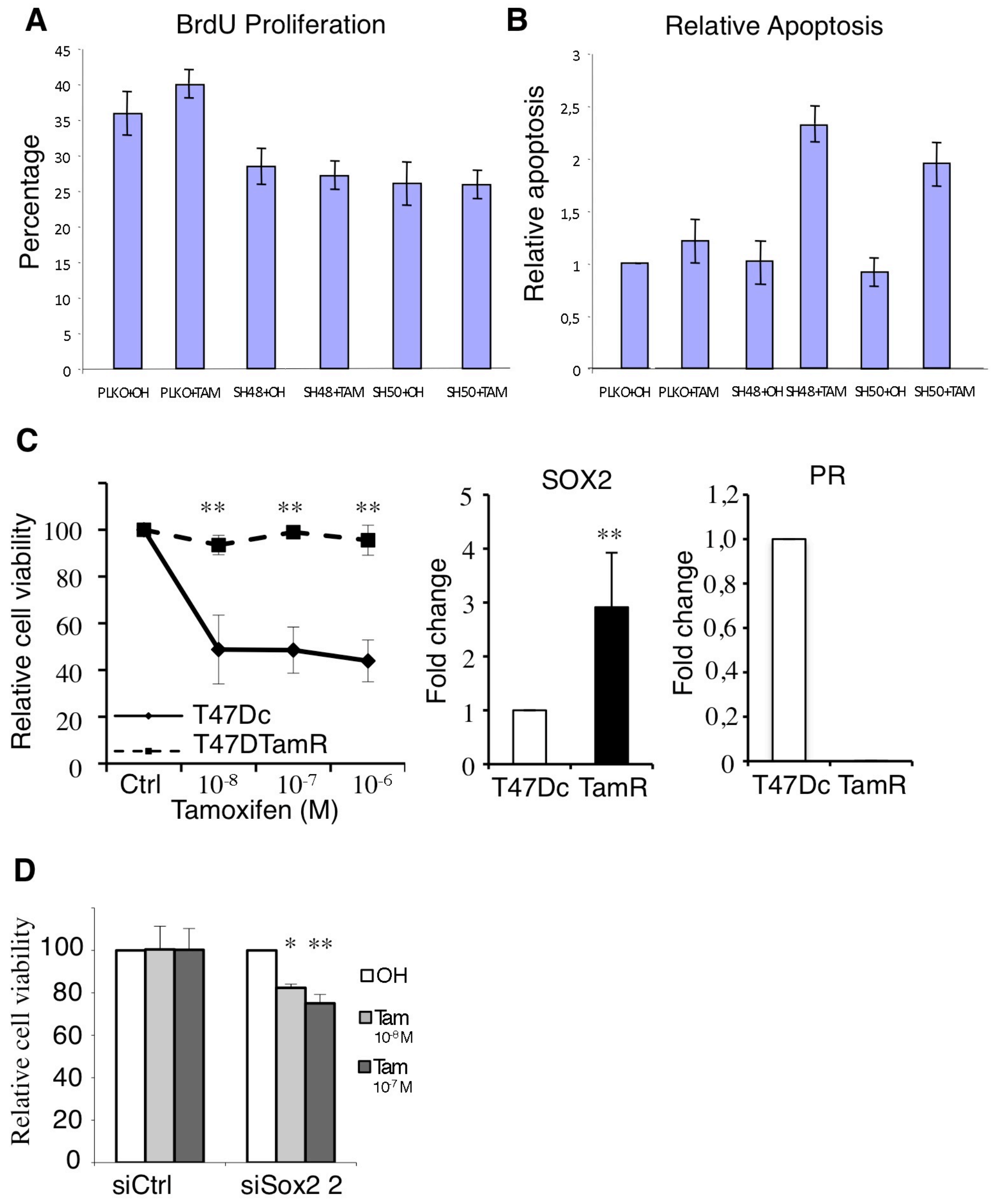
A
BrdU Proliferation
Percentage
PLKO+OH
PLKO+TAM
SH48+OH
SH48+TAM
SH50+OH
SH50+TAM
B
Relative Apoptosis
Relative apoptosis
C
Relative cell viability
T47Dc
T47DTamR
Tamoxifen (M)
Ctrl
SOX2
Fold change
T47Dc
TamR
PR
D
Relative cell viability
OH
Tam
siCtrl
siSox2 2

Sup. Figure 4. Suppression of Sox2 causes the resistant cells to undergo apoptosis. Characterisation of T47DTamR cells.

A. BrdU incorporation assay of control MCF7-TamR cells (shC = PLKO) and cells stably expressing reduced levels of Sox2, two different clones, sh48 and sh50, in the absence or presence of tamoxifen. Cells were incubated with BrdU during 6 hours following a 4-day period in the absence or presence of tamoxifen (n = 3). **B.** Cells with reduced Sox2 expression levels undergo apoptosis when treated with tamoxifen. Annexin V assay with different cells, as above, treated with 10^{-6}M tamoxifen during a period of 4 days. Experiments were performed three independent times. Error bars represent standard deviation. **C.** Characterisation of T47DTamR cells. Left: Proliferation assay (crystal violet) of parental T47D (T47Dc) and tamoxifen resistant (T47DTamR) cells (n = 3) $^{**}p = 0.009$, 0.008, 0.008, respectively, by t-test. Cells were treated with the vehicle ethanol (Ctrl) or increasing concentration of tamoxifen during a period of 4 days. Transcript levels of SOX2 (middle) and PR (right) in parental T47D (T47Dc) and T47DTamR (TamR) cells were quantified by real-time PCR and presented as fold induction with T47Dc value set as 1 (n = 3) $^{**}p = 0.0056$ by t-test. **D.** Viability assays (crystal violet) of T47DTamR cells transfected with a control siRNA sequence (siCtrl) and a Sox2 siRNA sequence (siSox2 2) growing in the presence of vehicle (ethanol, OH) or tamoxifen at different concentrations (10^{-8} M, light grey bars, $^{*}p = 0.04$ or 10^{-7} M, dark grey bars, $^{**}p = 0.01$ by t-test, tamoxifen) (n = 3).
