# Supplementary material for: Sox2 promotes tamoxifen resistance in breast cancer cells
Source: EMBO Mol Med. 2013 Oct 31;6(1):66–79. doi: 10.1002/emmm.201303411 (PMC3936493; doi:10.1002/emmm.201303411)
Supplement: Supplementary file 6 [file emmm0006-0066-sd6.pdf]

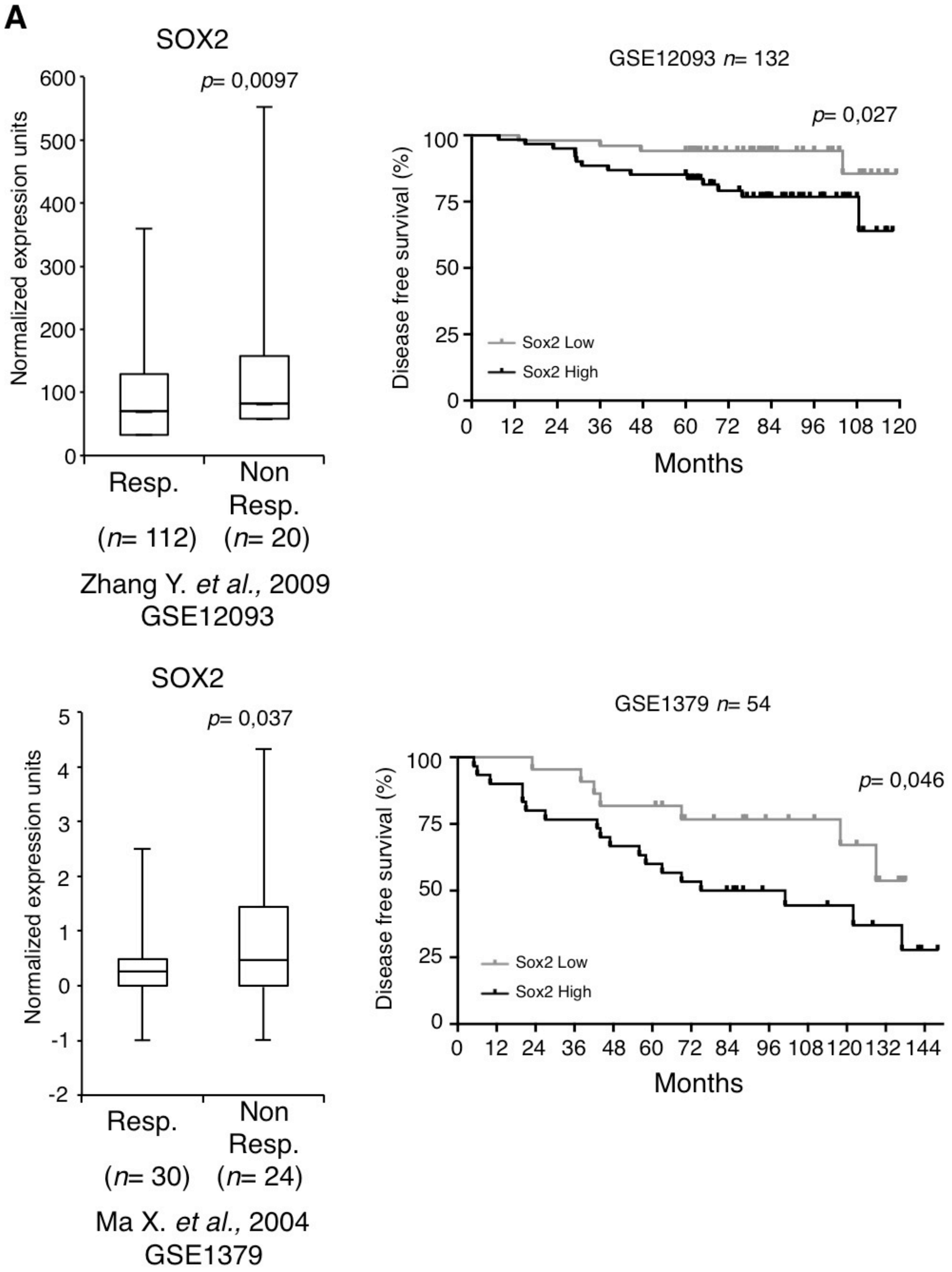
A
SOX2
p= 0,0097
Normalized expression units
600
500
400
300
200
100
0
Resp.
Non Resp.
(n= 112)
(n= 20)
Zhang Y. et al., 2009
GSE12093
GSE12093 n= 132
p= 0,027
Disease free survival (%)
Sox2 Low
Sox2 High
0 12 24 36 48 60 72 84 96 108 120
Months
SOX2
p= 0,037
5
4
3
2
1
0
-1
-2
(n= 30)
(n= 24)
Ma X. et al., 2004
GSE1379
GSE1379 n= 54
p= 0,046
0 12 24 36 48 60 72 84 96 108 120 132 144

Sup. Figure 5. Sox2 prognostic relevance.

A. Correlation between Sox2 expression and recurrence of ER-positive tamoxifen treated primary breast cancer samples. (Left) Two different data sets demonstrate similar correlation between increased SOX2 mRNA expression and therapy failure (Zhang Y. *et al.,* 2009 and Ma X. *et al.,* 2004). Box plots from the studies indicated are shown. The *y* axis shows normalised expression units. All data are median centered and the 25th–75th percentiles are indicated by the closed box. (Right) The association between SOX2 expression levels and disease free survival was evaluated by Kaplan–Meier analysis. The Sox2 low group was defined by having Sox2 expression lower than the median of all patients in the study, and the rest of patients were of the Sox2 high group. The number of breast carcinoma samples present in each group is shown within parenthesis and GEO accession number of each date set is indicated. Non Resp, non-responder tumours; Resp, responder tumours.

References

Ma XJ, Wang Z, Ryan PD, Isakoff SJ, Barmettler A, Fuller A, Muir B, Mohapatra G, Salunga R, Tuggle JT, Tran Y, Tran D, Tassin A, Amon P, Wang W, Wang W, Enright E, Stecker K, Estepa-Sabal E, Smith B, Younger J, Balis U, Michaelson J, Bhan A, Habin K, Baer TM, Brugge J, Haber DA, Erlander MG, Sgroi DC. (2004) A two-gene expression ratio predicts clinical outcome in breast cancer patients treated with tamoxifen. *Cancer Cell*, **5**, 607-616.

Zhang Y, Sieuwerts AM, McGreevy M, Casey G, Cufer T, Paradiso A, Harbeck N, Span PN, Hicks DG, Crowe J, Tubbs RR, Budd GT, Lyons J, Sweep FC, Schmitt M, Schittulli F, Golouh R, Talantov D, Wang Y, Foekens JA. (2009) The 76-gene signature defines high-risk patients that benefit from adjuvant tamoxifen therapy. *Breast Cancer Res Treat*, **116**, 303-309.
