# Supplementary material for: Sox2 promotes tamoxifen resistance in breast cancer cells
Source: EMBO Mol Med. 2013 Oct 31;6(1):66–79. doi: 10.1002/emmm.201303411 (PMC3936493; doi:10.1002/emmm.201303411)
Supplement: Supplementary file 7 [file emmm0006-0066-sd7.pdf]

A

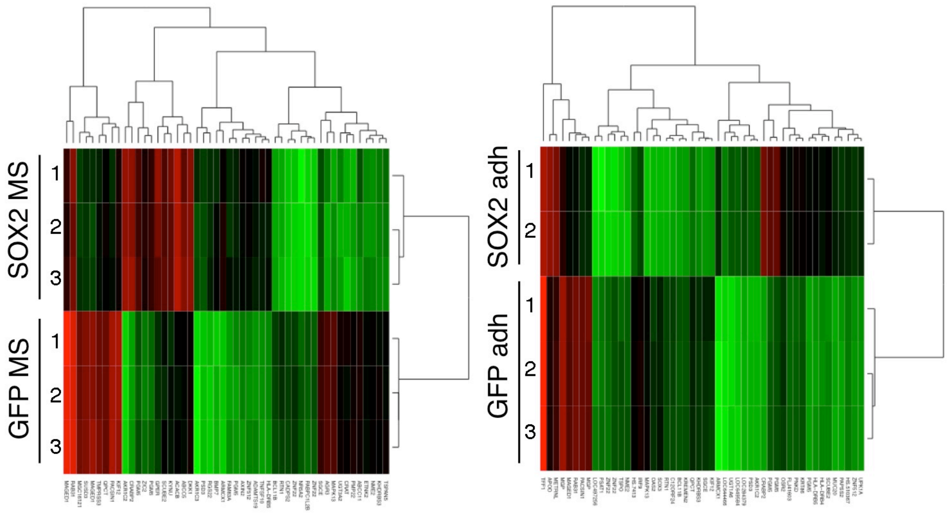

B

**Response to drug**	CDK ADD3, PLIN2, COMT, CTPS, EDN1, EMX1, ERBB2, ERBB3, ABCA3, ALDH1A3, FOS, AMH, GNAS, GPX3, HDAC2, HMBS, HMGCS1, IGFBP2, KCNJ11, RHOC, LGALS1, LRP2, MAP1B, ASS1, MYC, NME1, PNP, GAL, PIK3R1, BAK1, BCL2, SLC9A1, SRC, SST, THBS1, TSPO, CA4, BCAR3, RAD54L, CAV1, SOCS1
**Steroid hormone mediated signalling pathway**	PGRMC2, PGR, PPARD, BMP4, BMP7
**Response to estrogen stimulus**	CTNNA1, CYP27B1, CRIPAK, KRT19, STS, GAL, BCL2, TIMP3, CA2, CAV1, ASH2L
**Wnt signalling**	DKK1, AXIN2, FZD4, WNT7B, FZD2, TCF7L1, BTRC, ROR2, CXXC4, KREMEN2, CTGF

C

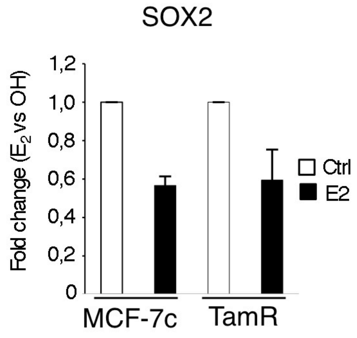

D

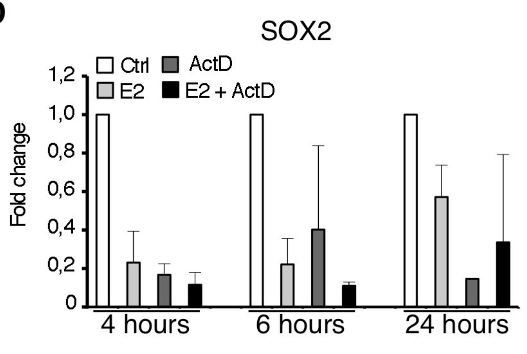

E

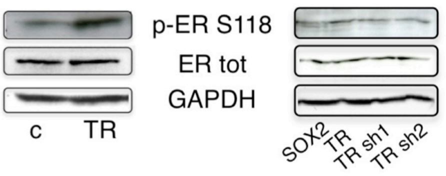

F

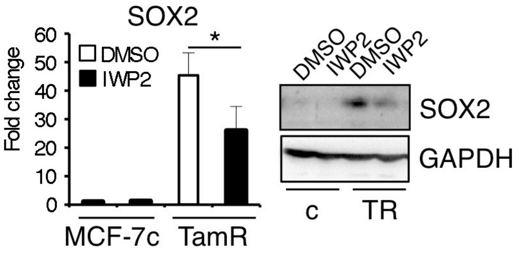

Sup. Figure 6. Gene expression profile of Sox2 overexpressing cells.

A. Heat maps of the 50 most significantly regulated genes in MCF7 cells overexpressing SOX2 versus the control cells grown as mammospheres (left) or in adherent conditions (right). **B.** Differently regulated genes in adherent MCF-7SOX2 cells involved in important pathways for breast cancer stem cells. **C.** Quantitative PCR analysis of SOX2 mRNA expression levels in control (MCF-7c) and resistant (TamR) cells treated with vehicle ethanol (Ctrl, white bars) or estrogen (10 nM, black bars) during 6 hours (n = 3). **D.** Quantitative PCR analysis of SOX2 mRNA expression levels in MCF-7TamR cells treated with vehicle ethanol (Ctrl), actinomycin D (0,5 μg/ml) at the indicated times, estrogen (10 nM), or both together (n = 3). **E.** Immunoblotting of parental (MCF-7c) and tamoxifen resistant (TamR) cells for P-S118, total ERα, and β–actin as control (left) and of MCF-7 cells overexpressing Sox2 (SOX2), MCF-7TamR cells stably transfected with control sHRNA sequences (shC) or two different specific shRNA sequences (sh1 and sh2) (right). **F.** Sox2 expression levels in parental MCF-7 (c) or MCF-7TamR cells (TR) treated with vehicle (DMSO) or the Wnt inhibitor IWP2 (2 μM) during a period of 2 days determined by Western blot (Top) or qPCR analysis (Bottom) (n = 3) $*p = 0.04$ by t-test.
