# Supplementary material for: Sox2 promotes tamoxifen resistance in breast cancer cells
Source: EMBO Mol Med. 2013 Oct 31;6(1):66–79. doi: 10.1002/emmm.201303411 (PMC3936493; doi:10.1002/emmm.201303411)
Supplement: Supplementary file 8 [file emmm0006-0066-sd8.pdf]

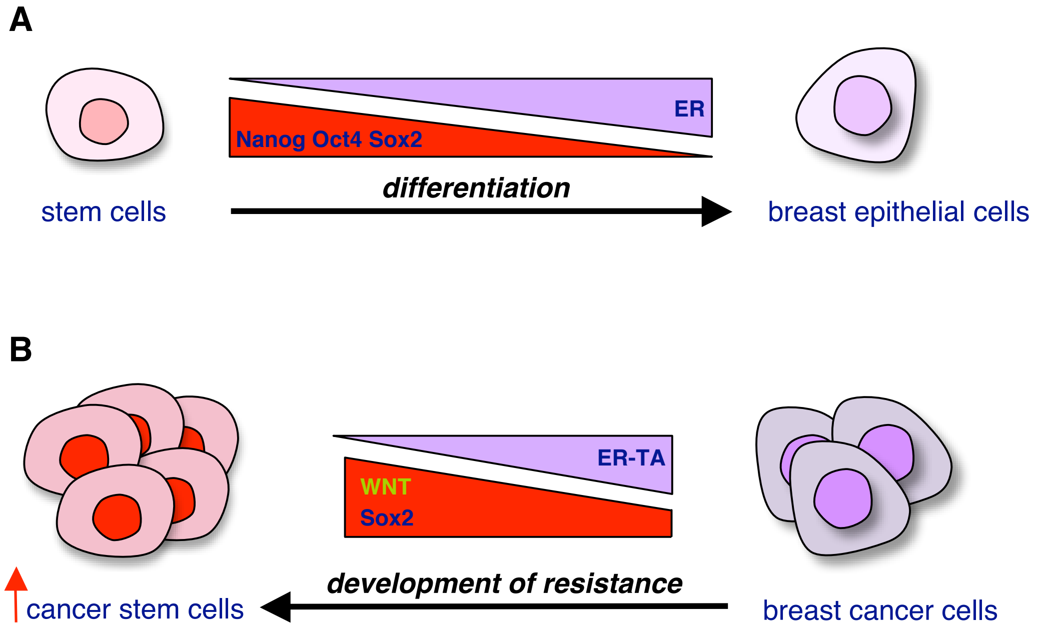

Figure 7. Development of resistance to tamoxifen reverts tumour cells to a more undifferentiated phenotype.

A. Differentiation to epithelial cells in the normal breast. ER expression is absent in breast stem cells and Nanog, Oct4 and Sox2 expression is downregulated during differentiation. **B.** Sox2 promotes tamoxifen resistance in breast tumour cells. ER transcriptional activity (ER-TA) is lost and Sox2 expression is increased further during development of resistance leading to Wnt signalling activation and increased cancer stem cell population.
