# Supplementary material for: Sox2 promotes tamoxifen resistance in breast cancer cells
Source: EMBO Mol Med. 2013 Oct 31;6(1):66–79. doi: 10.1002/emmm.201303411 (PMC3936493; doi:10.1002/emmm.201303411)
Supplement: Supplementary file 9 [file emmm0006-0066-sd9.pdf]

Table1: Correlations between Sox2 expression and clinicopathological and immunohistochemical features in breast carcinomas.

	Sox2 negative *n*= 33	Sox2 positive *n*= 48	p value (χ^2)
Size			0.52
T1	14/33	12/30	
T2	17/33	18/30	
Histol. Grade			0.006
G1	4/33	4/48	
G2	25/33	26/48	
G3	2/33	18/48	
Age			0.64
<50	10/33	8/48	
>50	23/33	32/48	
HER2			0.19
Positive	5/33	8/44	
Negative	28/33	34/44	
P53			0.09
Positive	13/33	18/43	
Negative	20/33	24/43	
