# Supplementary material for: Sox2 promotes tamoxifen resistance in breast cancer cells
Source: EMBO Mol Med. 2013 Oct 31;6(1):66–79. doi: 10.1002/emmm.201303411 (PMC3936493; doi:10.1002/emmm.201303411)
Supplement: Supplementary file 10 [file emmm0006-0066-sd10.pdf]

SUPPORTING MATERIALS AND METHODS

Cell culture and establishment of TamR cells

As it was done with MCF-7 cells, T47D cells were cultured in DMEM:F12 medium with GlutaMAX (Gibco) supplemented with 8% foetal bovine serum, FBS, (Sigma) and 1% penicillin/streptomycin (Sigma) at 37ºC in 5% CO_2. Cells were grown in the presence of ethanol, as vehicle, and 5×10^{-7} M 4-OH-tamoxifen (Sigma), respectively, in DMEM:F12 with 8% FBS for 6 months. During this time the medium was replaced every 3 days and the cell cultures were passaged by trypsinization after 70-80% confluency was reached. During the first few weeks cell growth rates were strongly reduced by tamoxifen treatment (no effect was ever detected by the very small dose (<0.01% v/v) of ethanol provided to the T47D control cells. Eventually, cell growth gradually increased, leading to the development of the tamoxifen resistant cell line T47DTamR. These cells were maintained in culture with 4-OH-tamoxifen for a further 2 months before characterization. T47Dc (parental control) and T47DTamR have been routinely maintained in the presence of ethanol, as vehicle, and 5×10^{-7} M of 4-OH-tamoxifen, respectively. Their cell growth properties have remained stable since then.

Mammosphere culture

For mammosphere culture, cells were plated in 75 cm^2 poly-HEMA (Sigma) coated plates at 5.000 cells/ml. Suspension cells were cultured in DMEM:F12 medium with GlutaMAX supplemented with B27 (Gibco), 10 ng/ml EGF (Invitrogen), 2 ng/ml bFGF (Invitrogen) and 1% of penicillin/streptomycin at 37ºC in 5% CO_2. After 7 days cells were collected by centrifugation (500 g) and dissociated mechanically and

enzymatically using TrypLE (Invitrogen) to obtain single cell suspensions, and the cells were counted and plated again in suspension to originate secondary or tertiary mammosphere cultures.

Real-Time Polymerase Chain Reaction (PCR)

Real-time PCR was performed using the iTaq™ SYBR® Green Supermix with ROX (BioRad). cDNA was amplified using the following conditions: 95ºC for 5 min, 40 cycles of amplification (95ºC for 15 sec, 61ºC for 1 min) and dissociation stage. 36B4 was used as a reference transcript for normalization. The results are presented as fold change calculated with $2^{-\Delta\Delta ct}$ method. The sequences of the primers are the following:

Gene	FWD 5'-3'	REV 5'-3'
36B4	GTGTTCGACAATGGCAGCAT	GACACCCTCCAGGAAGCGA
ALDH1A3	TCTCGACAAAGCCCTGAAGT	TATTCGGCCAAAGCGTATTC
AXIN2	AAGTGCAAACTTTCGCCAAC	ACAGGATCGCTCCTCTTGAA
DKK1	ATGCGTCACGCTATGTGCT	TCTGGAATACCCATCCAAGG
FZD4	GACAACTTTCACACCGCTCA	TCTTCTCTGTGCACATTGGC
NANOG	CAGCTGTGTGTACTCAATGATAGATTT	ACACCATTGCTATTCTTCGGCCAGTTG
OCT4	GACAACAATGAAAATCTTCAGGAG	CTGGCGCCGGTTACAGAACCA
SOX2	GCACATGAACGGCTGGAGCAACG	TGCTGCGAGTAGGACATGCTGTAGG
WNT3A	GTGGAACTGCACCACCGT	ATGAGCGTGTCACTGCAAAG
WNT4	AAGGCCATCCTGACACACATG	GCTAGGCTCCAAGTACACCAGG

Invasion assay

In vitro invasion and migration assays were performed in a 24-well BD Falcon™ HTS Multiwell Insert System containing an 8 μm pore size PET (PolyEthylene Terepthalate) membrane. For invasion assays, the top of the upper wells was coated with 2 μg of Matrigel Basement Membrane Matrix (BD) diluted in 50 μl of DMEM:F12 medium and allowed to air-dry overnight. The following day the Matrigel was re-hydrated with medium for 2-3 h, and 100,000 cells, previously starved in serum-free medium for 24 h, were added to the upper chamber. The lower

chamber was filled with chemoattractive medium, containing 300 μl of medium supplemented with 20% FBS and 300 μl of conditioned medium (0.45 μm filtered) obtained from MCF-7 cells. Medium alone was used as a negative control. After 72 h of incubation, cells on the upper surface of the membrane were removed mechanically by wiping with a cotton swab, and the cells remaining on the lower side of the membrane were fixed and stained with crystal violet solution. For chemotaxis assays the same procedure was followed but the upper wells were not coated with Matrigel. At least nine different fields (400x magnification) from each well were counted to determine the number of invading cells. Mammospheres were starved by adding 1 volume of medium, without supplements, to the culture 24 h before adding 50.000 of sorted cells to the upper chamber.

Fluorescence activated cell sorting (FACS)

Luminal cells were visualized using the antibody ICR-2 (ICR, Sutton), a rat monoclonal antibody against EMA, and the secondary antibody goat anti-rat FITC (fluorescein isothiocyanate) (Southern Biotech); myoepithelial cells were labelled with a mouse phycoerythrin (PE) anti-CALLA antibody (DAKO, clone SS2/36). Cells were labelled with both primary antibodies added concurrently, followed by incubation with the secondary FITC antibody. Controls included incubation of cells with primary isotype control antibodies, namely rat IgG2α isotype antibody (DAKO), followed by incubation with secondary rabbit anti-rat FITC antibody and PE conjugated non-specific mouse IgG1κ antibody (DAKO). TO-PRO-3 (Molecular Probes) was added for dead cell exclusion. A single cell suspension was blocked for 15 min at room temperature in PBS plus 40% FBS and incubated with primary antibodies. Control samples were stained with PE conjugated non-specific mouse

IgG2a,ϰ antibody (BD) and APC-conjugated non-specific mouse IgG2b,κ antibody (BD) for CD24 and CD44, respectively. The viability dye Via-probe (BD) was added for dead cell exclusion. Cells were sorted using a FACSAria (Becton Dickinson) or analyzed in a FACSCanto II (Becton Dickinson) flow cytometer and the data were analysed using the FACSDiva software.

ALDEFLUOR assay

The ALDEFLUOR assay was carried out according to manufacturer's (Stemcell Technologies) guidelines. Briefly, dissociated single cells were suspended in Aldefluor assay buffer containing an ALDH substrate, bodipyaminoacetaldehyde (BAAA) at 1.5 mM and incubated for 45 min at 37ºC. A fraction of cells was incubated under identical conditions in the presence of a 2-fold molar excess of the ALDH inhibitor, diethylaminobenzaldehyde (DEAB). Cells were analyzed using a FACSAria or FACSCanto II (Becton Dickinson) flow cytometre and analyzed using the FACSDiva software.

MTT assay

MTT [3-(4,5-dimethylthiazol-2-yl)-2,5-diphenyltetrazolium bromide] assays were used to determine the rate of cell viability. MCF-7OH, MCF-7TamR, MCF-7 GFP and MCF-7 SOX2 cells were seeded in 96-well plates at a density of 1,000 cells per well. After 24 h 4-OH-tamoxifen was added in four replicates for concentration for each cell line. Medium was changed after 3 days and cell vitality was measured after 6 days. 15 µl MTT (at 5 mg/ml) was added to each well at a final concentration of 500 μg/ml, the mixture was further incubated for 4 h, and the liquid in the wells was removed thereafter. 120 µl of dimethyl sulfoxide were then added to each well, and

the absorbance was read with a UV SpectramaxM2 reader (Molecular Devices) at 550 nm. The relative cell viability was expressed as a percentage of the control that was not treated with 4-OH-tamoxifen. MCF-7OH and MCF-7TamR cells were plated as described above. After 24 h, IWP2 (Calbiochem) or the vehicle DMSO were added at the concentration indicated in Fig 7. After 6 h the medium was changed and IWP2, 10^{-7} M 4-OH-tamoxifen and 100 ng/ml recombinant WNT3A (R&D Systems) were added and after 4 days cell vitality was measured as described above. Fresh IWP2 and WNT3a were added every day. The relative cell viability was expressed as a percentage of the control that was treated with vehicle solutions.

Crystal violet analysis

For cell proliferation analysis 10,000 cells per well were plated in a 12 well plate. After 24 h, 10^{-6} M 4-OH-tamoxifen was added in triplicate for each cell line. After 96 h, cell growth was measured by staining with crystal violet (0,1% in 20% methanol) for 30 min at room temperature. The precipitate was solubilised in 10% acetic acid and measured at 595 nm. The relative cell viability was expressed as a percentage of the control that was not treated with 4-OH-tamoxifen.

BrdU and Annexin V assays

Proliferation and apoptosis determination assays were performed using Fastimmune anti-BrdU antibody (BD) and the FITC Annexin V Apoptosis detection kit I (BD Pharmigen) following the recommendations of the manufacturer.

Immunohistochemistry

Immunohistochemical staining was performed using the Leica Bond-III and detection systems and buffers from Novocastra. Following a preheating step for antigen retrieval (Bond epitope retrieval solution I, 20 min, 100ºC), sections were washed (Bond wash solution) and incubated with anti-Sox2 antibody (1:100, Stemcell Technologies, cat nr 01438) for 20 min at room temperature. Peroxidase was blocked for 10 min, followed by HRP and DAB with hematoxyline for contrast. Negative controls included omission of the primary antibody and IgG-matched control antibody. Two pathologists reviewed the samples blindly and graded the nuclear staining for Sox2 depending on its intensity and the percentage of positive neoplastic cells according to the Allred score.

Microarray hybridization and analysis

MCF-7v and MCF-7SOX2 cells were grown in adherent conditions for 24 h and in suspension conditions for 7 days (the three replicates were processed as three independent experiments). RNA was extracted with the RNeasy Mini Kit (Qiagen), followed by digestion of genomic DNA using the RNase-free DNase-Set (Qiagen). RNA concentration was measured on Nanodrop (Thermo scientific) and quality was determined on an Agilent Bioanalyzer. RNA was labelled using the Illumina TotalPrep RNA Amplification Kit (Ambion). Crude data for the analysis were extracted with Illumina's "GenomeStudio" data analysis software. These data were analysed using the R/Bioconductor statistical computing environment (www.r-project.org, www.bioconductor.org). Using the lumi Bioconductor package, raw expression data were log2 transformed and quantile normalized. The data for the probes with a detection p-value higher or equal to 0.01 were excluded. Those detected with p-value lower than 0.01 in at least one array were accepted as significant. Thus,

the data for the probes that did not pass the test were filtered out and the genes they represented considered to be ”not expressed”.
